# Evolutionary diversification of epidermal barrier genes in amphibians

**DOI:** 10.1038/s41598-022-18053-7

**Published:** 2022-08-10

**Authors:** Attila Placido Sachslehner, Leopold Eckhart

**Affiliations:** Skin Biology Laboratory, Department of Dermatology, Medical University of Vienna, Vienna, Austria

**Keywords:** Molecular evolution, Differentiation, Herpetology

## Abstract

The epidermal differentiation complex (EDC) is a cluster of genes encoding components of the skin barrier in terrestrial vertebrates. EDC genes can be categorized as S100 fused-type protein (SFTP) genes such as *filaggrin*, which contain two coding exons, and single-coding-exon EDC (SEDC) genes such as *loricrin*. SFTPs are known to be present in amniotes (mammals, reptiles and birds) and amphibians, whereas SEDCs have not yet been reported in amphibians. Here, we show that caecilians (Amphibia: Gymnophiona) have both SFTP and SEDC genes. Two to four SEDC genes were identified in the genomes of *Rhinatrema bivittatum*, *Microcaecilia unicolor* and *Geotrypetes seraphini*. Comparative analysis of tissue transcriptomes indicated predominant expression of SEDC genes in the skin of caecilians. The proteins encoded by caecilian SEDC genes resemble human SEDC proteins, such as involucrin and small proline-rich proteins, with regard to low sequence complexity and high contents of proline, glutamine and lysine. Our data reveal diversification of EDC genes in amphibians and suggest that SEDC-type skin barrier genes have originated either in a common ancestor of tetrapods followed by loss in Batrachia (frogs and salamanders) or, by convergent evolution, in caecilians and amniotes.

## Introduction

The evolutionary transition from water to land was associated with the evolution of a proficient skin barrier in terrestrial vertebrates^1,2^. The barrier function of the skin depends largely on epithelial cells, i.e. keratinocytes, which proliferate in the basal layer of the epidermis and undergo differentiation in the suprabasal layers thereof^3^. Keratinocyte differentiation involves expression of high amounts of keratin intermediate filaments to enforce the cytoskeleton and other genes which serve specific barrier-associated functions, such as cross-linking of proteins, building intercellular connections and anti-microbial defense^2,4^.

The epidermal differentiation complex (EDC)^5–7^ is a cluster of genes which are expressed in the late phase of keratinocyte differentiation. The genes of the EDC can be categorized according to their exon–intron organisation and the domain structure of the encoded proteins. S100A genes, consisting of one non-coding and two protein-coding exons, are located at the borders of the EDC. The same exon–intron organization is found in genes encoding S100 fused-type proteins (SFTPs), whereas single-coding-exon EDC (SEDC) genes^8^ have one non-coding and one coding exon. S100A’s are involved in calcium-associated signal transduction^9^, whereas SFTP and SEDC genes encode components of proteinaceous intracellular structures that provide mechanical stability to the outermost, cornified cell layers of the epidermis. The EDC was originally identified in the human and mouse genomes^5–7^ and mutations in specific EDC genes, such as *filaggrin* (*FLG*) and *late cornified envelope* (*LCE*) *3B*/*3C* were found to be associated with prevalent skin diseases, atopic dermatitis and psoriasis, respectively^10,11^. Subsequently, EDC gene clusters were also identified in other mammals^12–14^, birds^8,12,15,16^, crocodiles^17^, turtles^18^, squamates^8,19^, sphenodonts^20^ and amphibians^21^. Among amphibians, only a small portion of the phylogenetic diversity of this clade was covered by previous research^22^ because genome sequences were available from no other amphibians than frogs (Anura). The new availability of genome sequences of caecilians and a salamander allows us to extend the comparative analysis of the EDC in amphibians in the present study.

Caecilians (Amphibia: Gymnophiona) represent a phylogenetic lineage of amphibians which have diverged approximately 320 million years ago from batrachians (frogs and salamanders)^22,23^. They are limbless, have a burrowing lifestyle and can be found in tropical regions of America, Africa and Asia. The outermost cell layer of the epidermis of caecilians is cornified and covered by a layer of mucus produced by abundant skin glands^24,25^. In addition to these features shared with other amphibians, caecilian skin contains mineralized dermal scales^26^. Recently, genome sequences of three species of caecilians, i.e., the two-lined caecilian (*Rhinatrema bivittatum*), the Gaboon caecilian (*Geotrypetes seraphini*) and a representative of the family Siphonopidae (*Microcaecilia unicolor*), were reported^27–29^. However, genes regulating epidermal differentiation and particularly the EDC have not yet been characterized in caecilians.

Here, we analyzed the gene composition of the EDC of caecilians and compared it to the EDC of other amphibians and humans. We show that the EDC of caecilians differs from that of frogs and salamanders by the presence of an EDC gene class previously identified only in amniotes.

## Results

### Comparison of the EDC in caecilians, other amphibians and amniotes

**Figure 1:**
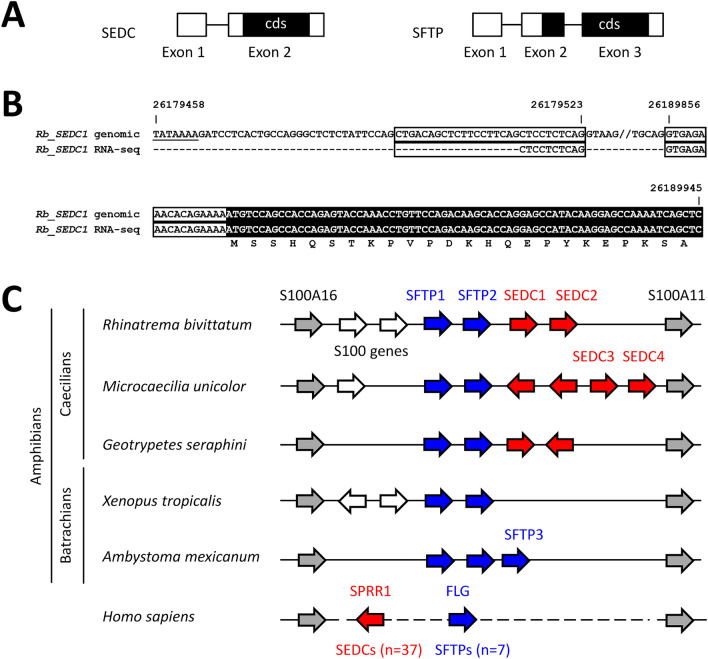
The EDC of caecilians contains SEDC genes. (**A**) Exon–intron structures of SEDC (single-coding-exon EDC) and S100 fused-type protein (SFTP) genes. Exons are shown as boxes in which the protein coding sequence (cds) is shaded black. (**B**) Alignment of the nucleotide sequences of the SEDC1 gene of *Rhinatrema bivittatum* (Rb) (GenBank accession number NC_042630.1, nucleotide positions are indicated above the sequence) and an RNA-seq read from the skin of this species (GenBank accession number: SRR5591419, experiment SRX2848310, read: gnl|SRA|SRR5591419.19486855.2). The TATA box is underlined. Only the first and last 5 nucleotides of the intron are shown. The sequence gap is indicated by “//”. Non-coding sequences of exons are indicated by a black box and coding sequences are shown with white fonts on black background. The amino acid sequence of the translation product is shown underneath the nucleotide sequence. (**C**) Structure of the EDC in amphibians in comparison to the human EDC. The genes of the chromosomal segments bordered by the conserved genes *S100A11* and *S100A16* in *Rhinatrema bivittatum* (two-lined caecilian), *Microcaecilia unicolor* (a common caecilian from French Guayana), *Geotrypetes seraphini* (Gaboon caecilian), *Xenopus tropicalis* (tropical clawed frog), *Ambystoma mexicanum* (axolotl) and *Homo sapiens* (human) are schematically depicted by arrows pointing in the direction of gene transcription. SEDC genes are shown as red arrows. SFTP genes are shown as blue arrows. Grey arrows indicate *S100A11* and *S100A16* genes. White arrows indicate other S100 genes located between *S100A11* and *S100A16* in amphibians. The dashed line in the human EDC indicates that only a subset of SEDC and SFTP genes and no other S100A genes are shown for lack of space. The total numbers of human SEDC and SFTP genes are indicated.

The EDC of three caecilians, *Rhinatrema bivittatum*, *Microcaecilia unicolor* and *Geotrypetes seraphini*, and two batrachians, the tropical clawed frog (*Xenopus tropicalis*) and the axolotl (*Ambystoma mexicanum*) was investigated for the presence of *SFTP* and *SEDC* genes. To identify *SFTP* and *SEDC* homologs, we followed a published approach involving tBLASTn searches using the S100 domains as queries for *SFTP* genes and de novo gene prediction from nucleotide sequences for finding *SEDC*’s^8^. The strategy is outlined in detail in the “Materials and methods” section. *SEDC* and *SFTP* genes were defined by their characteristic exon–intron structures with *SEDC* genes being comprised of a non-coding and a protein-coding exon and *SFTP* genes contain a non-coding and two coding exons (Fig. 1A). The presence of a non-coding exon upstream of the exon containing the coding sequence was confirmed by the identification of exon-spanning RNA-seq reads in the skin transcriptomes of caecilians (Fig. 1B). The location of the predicted *SFTP* and *SEDC* genes are summarized in Tables S1 and S2. Amino acid sequences of SFTP and SEDC proteins encoded by these genes are shown in Supplementary Fig. S1.

*SFTP* genes were identified in all species of amphibians investigated, whereas *SEDC*s were found only in caecilians. Two *SEDC* genes are present in *Rhinatrema bivittatum* and *Geotrypetes seraphini,* and 4 *SEDC*s are present in *Microcaecilia unicolor* (Fig. 1C). Each of the 3 species of caecilians investigated and *Xenopus tropicalis* have 2 *SFTP* genes. Three *SFTP*s were identified in the axolotl (Fig. 1C).

The locus of *SEDCs* in caecilians is not syntenic with that of *SEDC*s in humans and other amniotes^19^, because *SEDC*s of caecilians are located between *S100A16* and *SFTP* genes, whereas *SEDC*s of amniotes are located between the *SFTP* genes and *S100A11* (Fig. 1C).

### SFTP and SEDC genes of caecilians are expressed at higher levels in the skin than in other organs

**Figure 2:**
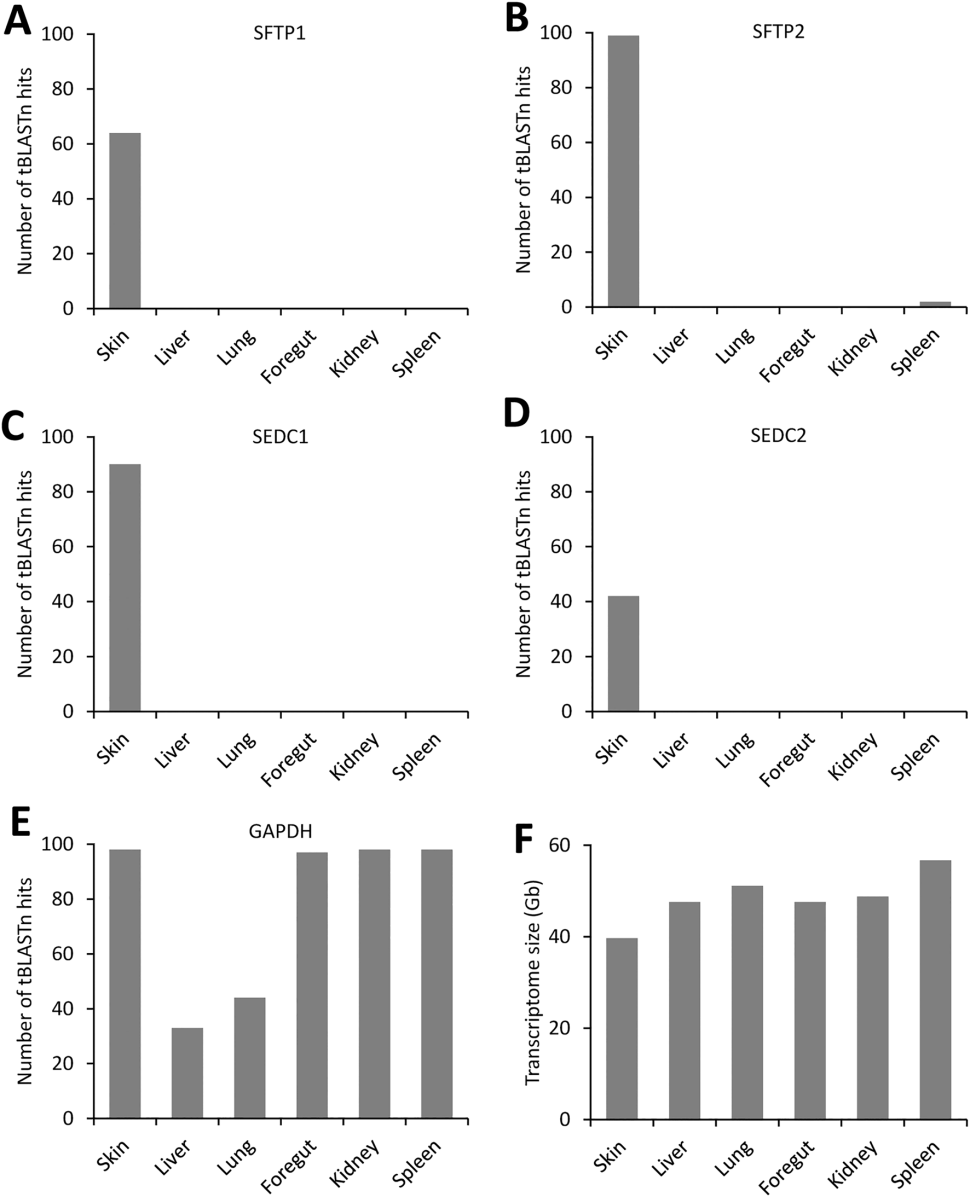
Semiquantitative analysis of EDC gene expression in tissue transcriptomes of *Rhinatrema bivittatum*. (**A**–**E**) Sequence fragments of the predicted proteins as described in the “Methods” section were used as queries for tBLASTn analysis. The accession numbers for the transcriptomes were as follows: skin (SRX2848310), liver (SRX2848294), lung (SRX2848293), foregut (SRX2848291), kidney (SRX2848286), spleen (SRX2848287). Default settings of tBLASTn were used except for deactivation of the filter for low complexity regions. tBLASTn hits with 100% sequence identity to the query were counted. *GAPDH* was investigated as a house-keeping gene (**E**). Note that this analysis allows comparison of expression levels of a particular gene in different organs, but not comparison between expression levels of different genes. (**F**) Size of tissue transcriptomes. *Gb* Gigabases.

To compare the expression levels of EDC genes in different organs of caecilians, we performed a tBLASTn search for EDC genes in the transcriptomes of the organs of *Rhinatrema bivittatum*. SFTP1 (Fig. 2A), SFTP2 (Fig. 2B), SEDC1 (Fig. 2C) and SEDC2 (Fig. 2D) were abundantly detected in the skin but not, or only at minute levels, in other organs whereas a house-keeping gene was detected at similar levels in all organs (Fig. 2E). As the transcriptomes of all organs contain comparable numbers of RNA-sequencing reads (Fig. 2F), we conclude that EDCs genes of *Rhinatrema bivittatum* are predominantly expressed in the skin.

### SEDC proteins of caecilians have high contents of proline, glutamine and lysine

**Figure 3:**
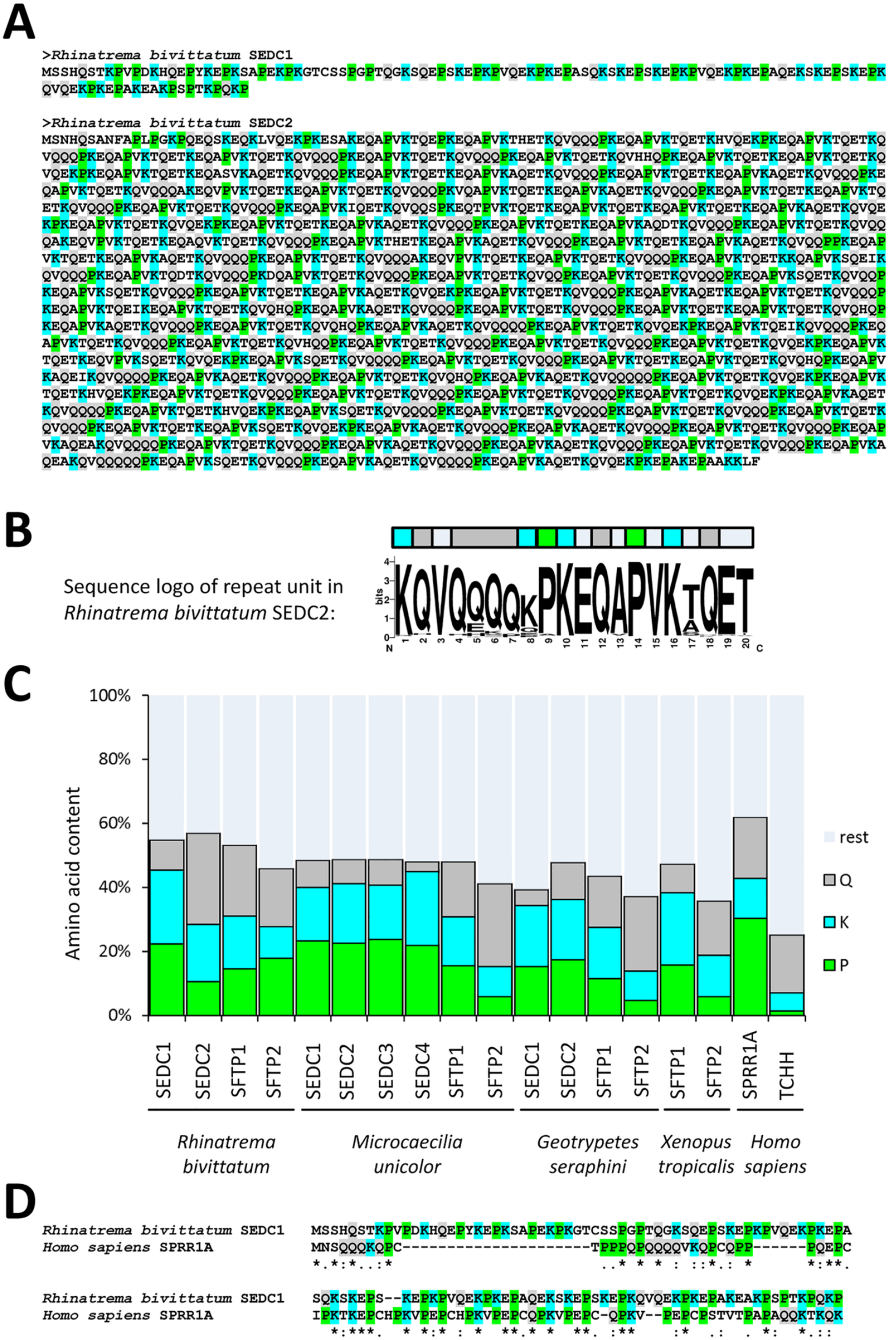
Amino acid sequences of caecilian EDC proteins are rich in glutamine, lysine and proline. (**A**) Amino acid sequences of *Rhinatrema bivittatum* SEDC proteins. (**B**) Sequence logo of the repeat unit in *Rhinatrema bivittatum* SEDC2 based on the alignment of repeats shown in Supplementary Fig. S2. (**C**) Amino acid contents of EDC proteins of caecilians and *Xenopus tropicalis*. For comparison, the values for two human EDC proteins (SPRR1A, an SEDC protein, GenBank accession NP_001186757.1, and trichohyalin, TCHH, an SFTP, GenBank accession number NP_009044.2) are shown on the right. (**D**) Amino acid sequence alignment of *Rhinatrema bivittatum* SEDC1 and human SPRR1A. Glutamine, lysine and proline residues are highlighted by grey, blue and green shading, respectively.

The amino acid sequences of SEDC proteins of caecilians are characterized by low sequence complexity (Fig. 3A, Supplementary Fig. S1) and the presence of sequence repeats (Fig. 3B, Supplementary Fig. S2). The amino acid contents of both SEDC and SFTPs of caecilians as well as those of SFTPs of *X. tropicalis* are biased towards proline, glutamine and lysine (Fig. 3C). Proline disrupts or terminates secondary structures in proteins, and glutamine and lysine are the targets of transglutamination, a process critical for epidermal cornification^4^. In addition, all three amino acids are associated with a high propensity to form disordered protein segments^30^, suggesting that SEDC proteins and the carboxy-terminal domain of SFTPs of caecilians are intrinsically disordered proteins, thus resembling their counterparts in mammals^31–33^. Sequence alignment showed similarities between SEDC proteins of caecilians and small proline-rich proteins (SPRRs) of mammals (Fig. 3D). However, the sequence similarity was largely due to the similarly biased amino acid composition of these proteins and therefore did not represent strong evidence for homology. For this reason, amino acid sequence alignments of SEDC proteins were highly ambiguous, and we refrained from building phylogenetic models based on these alignments. By contrast, the sequences of the S100 domains at the amino-terminus of SFTPs could be aligned well, and molecular phylogenetics supported shared ancestry of SFTPs of amphibians and amniotes (Figs. S3, S4).

## Discussion

**Figure 4:**
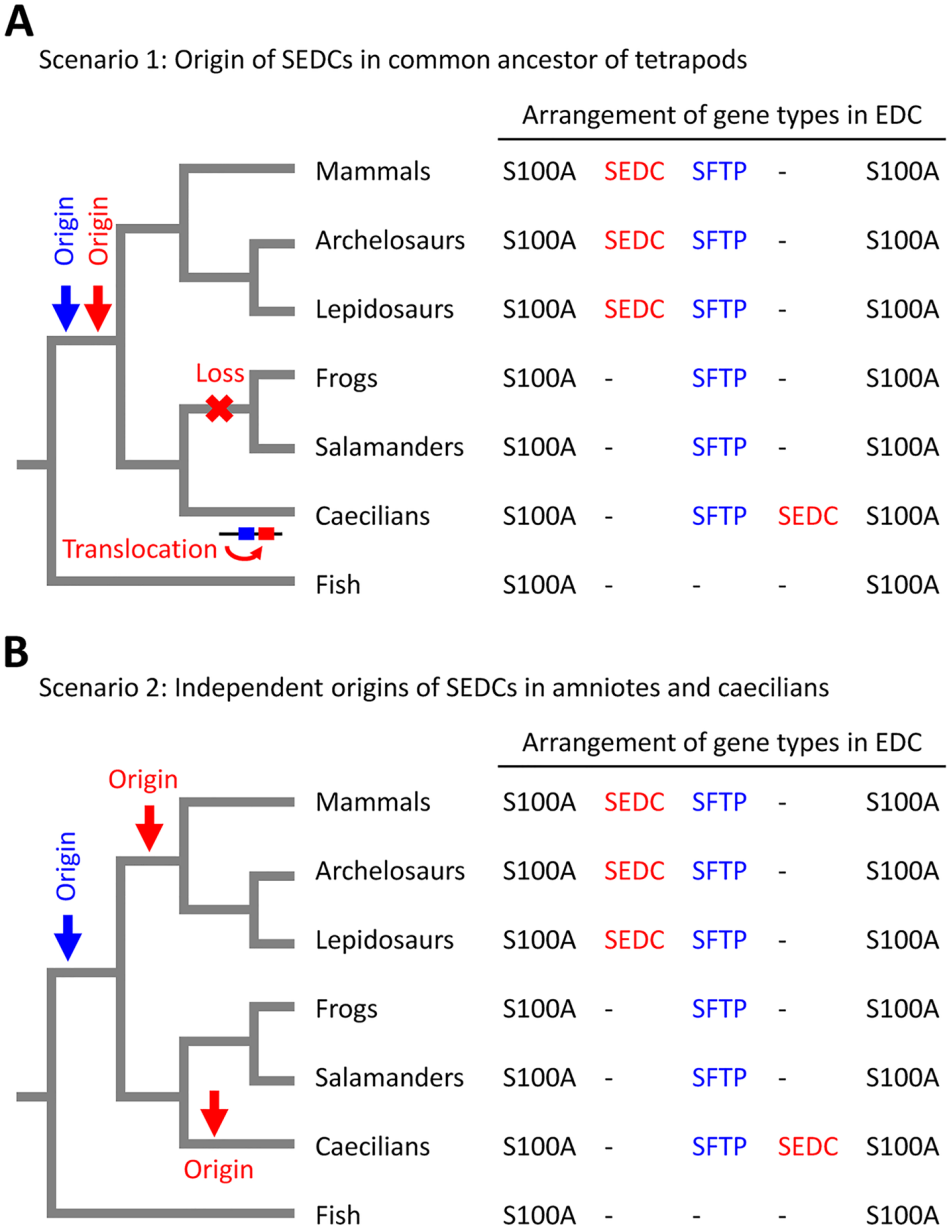
Scenarios for the evolution of SEDC genes in tetrapods. Two alternative scenarios (**A**, **B**) for the evolution of SEDC genes in terrestrial vertebrates are schematically depicted. Both scenarios are compatible with the arrangement of gene types in the epidermal differentiation complex (EDC) of caecilians and other main taxa of vertebrates. The relative arrangement of SEDC, SFTP and S100A genes within the EDC of each taxon is indicated on the right.

The results of this study show that the organization of the EDC of caecilians (Gymnophiona) differs from that of representatives of frogs (Anura) and salamanders (Caudata) as only caecilians have *SEDC* genes. This finding indicates that the evolution of *SEDC*s was more complex than that of *SFTP*s, which exist in all major taxa of terrestrial vertebrates but not in fish^21^, including lungfish (Supplementary Fig. S5), and therefore have evolved in a common ancestor of tetrapods^21^. To infer the evolutionary histories of *SEDC*s, we compared the presence or absence and the chromosomal arrangement of *SEDC*s relative to *SFTP* and *S100A* genes in the main taxa of terrestrial vertebrates (Fig. 4). The distribution of *SEDC* genes among modern amphibians and amniotes suggests two alternative evolutionary scenarios for the origin of *SEDC*s (Fig. 4). In scenario 1, *SEDC*s originated in a common ancestor of tetrapods and were conserved in amniotes and caecilians whereby a translocation of *SEDC* relative to *SFTP* genes occurred in either one of the two clades and the primordial *SEDC* gene was lost in batrachians (frogs and salamanders) (Fig. 4A). In scenario 2, *SEDC*s originated independently in the phylogenetic lineage leading to caecilians and in the phylogenetic lineage leading to amniotes (Fig. 4B). Due to the low sequence complexity of SEDCs, faithful sequence alignments required for phylogenetic analysis are not possible and it remains unclear which of the two scenarios is more likely to be correct. A hypothetical pathway of gene recombination and mutation events underlying an evolutionary trajectory from *S100A* to *SFTP* and *SEDC* genes is schematically depicted in Supplementary Fig. S6. Independently of the question about the relationship of *SEDC*s of caecilians and amniotes, the presence of SEDCs in caecilians demonstrates that the evolutionary diversification of epidermal barrier genes is not restricted to amniotes^8^, but has also occurred in the amphibian clade of terrestrial vertebrates.

Our finding that *SEDC* genes of caecilians are expressed in the skin but not, or only at low levels, in other organs, indicates that these EDC genes, like their counterparts in amniotes, are specifically important for the body’s barrier against the environment, i.e. the primordial function of the skin^3,34^. Each species of caecilians investigated has at least two *SEDC* genes with distinct amino acid sequences (Fig. 3A). Further studies are required to determine the expression pattern of individual *SEDC* genes in the various layers of the skin, during development of the skin and at different stages of the shedding cycle of the epidermis. It will be interesting to test whether individual *SEDC*s and *SFTP*s have unique or shared expression patterns in caecilians.

The amino acid sequences of SEDC proteins of caecilians resemble those of SPRRs and other proline-rich EDC proteins of amniotes^14,18^. The high contents of proline, glutamine and lysine indicate that these proteins lack secondary structures^30^, thus representing intrinsically disordered proteins. The high contents of glutamine and lysine suggest that SEDCs serve as substrates for transglutamination, the main function of involucrin, loricrin and other mammalian SEDCs during epidermal cornification^4^. Interestingly, *Rhinatrema* SEDC2 contains many stretches of 3–5 consecutive glutamine residues, thereby resembling human involucrin. Besides these sequence similarities, there are also important differences between SEDCs of caecilians and amniotes. Sequence motifs that are conserved at the amino- and carboxy-termini of many SEDCs of amniotes^8,17^, are not present in the SEDCs of caecilians. Furthermore, it is noteworthy that amniotes have a much greater diversity of SEDC proteins, including cornified beta-proteins (CBPs), also known as beta-keratins^8,35^, glycine-rich proteins such as loricrin^36^ and cysteine-rich proteins present in feathers^37,38^. The relatively low cysteine content of SEDC proteins of caecilians suggests that protein cross-linking through disulfide bonds is not critical for epidermal cornification in these amphibians. The limited number and sequence diversity of SEDCs of caecilians corresponds to the rather uniform morphology of the epidermis of caecilians relative to amniotes.

The cornification of the epidermis is of great importance for caecilians and other amphibians^39^. The cornified layer protects the body in a terrestrial environment, it serves in feeding offspring of the caecilian, *Boulengerula taitanus*^40^ and it is the site of infection with chytrid fungi, which are implicated in the global decline of populations of amphibians^41,42^. As the molecular basis for these functions and interactions of amphibian epidermis are largely unknown, the results of the present study contribute to a better understanding of the biology of amphibian skin.

## Methods

### Ethics statement

No studies involving live animals were performed. Genomes and transcriptomes of vertebrates were investigated exclusively using sequences available in public databases.

### Identification of EDC genes

EDC genes were identified in the genome sequences of *Rhinatrema bivittatum*, GenBank accession number NC_042630.1, *Microcaecilia unicolor*, GenBank accession number NC_044044.1, and *Geotrypetes seraphini*, GenBank accession number NC_047099.1, submitted by Wellcome Sanger Institute, Wellcome Genome Campus, Hinxton, Cambridge, UK (Tables S1, S2)^27–29^. The EDC was also analyzed in the genomes of the tropical clawed frog (*Xenopus tropicalis*, GenBank accession number NC_030684.2, submitted by University of California, Berkeley, CA, USA) and the axolotl (*Ambystoma mexicanum*, GenBank accession number CM010927.2, submitted by Max Planck Society/University of Kentucky, Lexington, KY, USA). Furthermore, the locus of S100 genes on chromosome 8 of the West African lungfish (*Protopterus annectens*) was analyzed (GenBank accession number NC_056741.1, whole-genome assembly released by the Northwestern Polytechnical University, Xian, China)^43^.

To identify EDC genes in the currently available genome sequences of amphibians, we followed a published protocol with modifications^8^. Briefly, the nucleotide sequence between the *S100A* genes, which are located on the borders of the EDC, was searched for *SFTP* and *SEDC* genes. *SFTP* genes were identified by tBLASTn search using the S100 domains of *Xenopus* SFTPs^21^ as queries. Genes in which the coding sequence extended for more than 400 nucleotides over the end of the S100 domain region without coding for another defined structural domain were classified as *SFTP* genes. Of note, genes encoding two consecutive S100 domains, such as the *S100A11* genes of *Rhinatrema bivitattum* and *Xenopus tropicalis*, were classified as *S100A* genes. Candidate *SEDC* genes were searched by translating the entire nucleotide regions located between *SFTP* genes and the next *S100A* gene on each side of the *SFTP* genes. Open reading frames encoding polypeptides of 50 or more amino acid residues with a composition biased towards either glycine and serine (loricrin-like), glutamine (involucrin-like), proline (SPRR-like) or cysteine (EDCRP-like) were analyzed further. The open reading frames were considered *SEDC*-like if they were preceded by a *bona fide* splice acceptor motif within 50 nucleotides upstream of the putative start codon. To test if the predicted genes are transcribed, we performed BLAST searches in transcriptomes of caecilians that were available in the single-read archive (SRA) of the GenBank. The non-coding exon 1 of *SFTP* and *SEDC* genes was identified by alignment of transcript reads against the genomic DNA upstream of the coding region, followed by verification of the presence of a TATA box and a splice donor sequence. Some exon–intron borders of EDC genes of caecilians were correctly predicted by the gene prediction algorithm of the GenBank. However, the protein-coding potential of most genes was not identified by this algorithm. Accordingly, we corrected gene predictions where necessary (Figs. S1, S2). Orthology of genes was assessed using the criteria of shared local synteny and best reciprocal sequence similarity in BLAST.

### Analysis of amino acid sequences encoded by EDC genes

The predicted EDC genes were translated with the Expasy Translate tool^44^ (https://web.expasy.org/translate/, last accessed on 3 March 2022). The resulting amino acids were aligned with MUSCLE, version 3.8.425 using default parameters at https://www.ebi.ac.uk/Tools/msa/muscle/ or MultAlin^45^ followed by manual optimization, highlighting of sequence motifs and repeats. Percentage content of amino acids for each predicted sequence was determined with the Expasy Protparam tool^44^ (https://web.expasy.org/protparam/, last accessed on 3 March 2022). Sequence logos were created with the Weblogo tool at https://weblogo.berkeley.edu/logo.cgi (last accessed on 3 March 2022).

### Analysis of EDC gene expression in tissues of *Rhinatrema bivittatum*

The expression of EDC genes was validated by tBLASTn searches in the tissue transcriptomes of *Rhinatrema bivittatum* (Skin: SRX2848310, liver: SRX2848294, lung: SRX2848293, foregut: SRX2848291, kidney: SRX2848286, spleen: SRX2848287). The following sequences were used as queries: *Rhinatrema bivittatum* SFTP1: amino acid positions 41–60, *Rhinatrema bivittatum* SFTP2: amino acid positions 1–40, *Rhinatrema bivittatum* SEDC1: amino acid positions 1–50, *Rhinatrema bivittatum* SEDC2: amino acid positions 41–60 (Supplementary Fig. S1), *Rhinatrema bivittatum* GAPDH (XP_029432574): amino acid positions 211–336. The tBLASTn search was run with default parameters, but the option “filter for low complexity regions” was not used. The number of hits in each tissue was determined, whereby only hits with 100% sequence identity to the query were counted.

### Molecular phylogenetics

Sequences of *SFTP* and *S100A16* genes for the phylogenetic analysis were downloaded from NCBI GenBank. The sequences were aligned using MAFFT (multiple alignment using fast fourier transform) (Version 7.427)^46^ with the parameters “-maxiterate” set to 1000 and “-localpair”. Sequences were trimmed manually with aliview^47^, and only the sequences of S100 domains were aligned for the phylogenetic analysis (Supplementary Fig. S3). The model for amino acid replacement was calculated using prottest (Version 3.0)^48^. All available matrices (setting “-all-matrices”) and models with rate variation among sites (setting “-all-distributions”) were included. The likelihood of the predicted models was assessed with the Akaike information criterion (setting “-sort A”)^49^. The selected amino acid substitution model for the SFTP phylogeny was LG^50^. Maximum likelihood tree and bootstrap analysis (setting “-b 100”) were performed using PHYML (Version 20120412). Tree topology, branch length, and rate parameters were optimized (setting “-o tlr”)^51^. Phylogenetic trees (Supplementary Fig. S4) were visualized and annotated with FigTree (http://tree.bio.ed.ac.uk/software/figtree/, last accessed on 3 March 2022).

## Supplementary Information

Supplementary Information.

## Data Availability

All data generated or analyzed during this study are included in this published article and its Supplementary Information files.
