# Supplementary material for: Evolutionary diversification of epidermal barrier genes in amphibians
Source: Sci Rep. 2022 Aug 10;12:13634. doi: 10.1038/s41598-022-18053-7 (PMC9365767; doi:10.1038/s41598-022-18053-7)
Supplement: Supplementary file 1 — Supplementary Information. [file 41598_2022_18053_MOESM1_ESM.pdf]

Supplementary Data: Supplementary Figures and Tables

Evolutionary diversification of epidermal barrier genes in amphibians

Attila Placido Sachslehner, Leopold Eckhart

Content

Supplementary Figures S1-S6

Supplementary Tables S1-S2

A

>Gs_SEDC1
MSHYQSTKSAPEKPQEAIKDPKPMHEKPKEAVKEPEHPKSHDSKSAADPVKHQDTSKDTKPVKEPEQPKSPCSKSAPIPVKNQEVSKHPESVHEKLK
EAVKEPEHPKSHDNKTAPVPVKHQDASKDPTPVKEQEQPKSSCSKSVPIPVKNQEASKDTKPVHEKLKEAVKEPEHPKSHDNKTAPVPVKHQDASKD
LKPVKEPEQPKSHDSKSAPIPVKNQEVPKDTKPVKEPEKPKSHDNKTAPVPVKHQDASKDLKPVKEPEQPKSHDSKSAPIPVKYQEVSKDTKPMQEK
PKEPEQPKSPCSKSAPIPVKHQEASTHPESVHEKLKEAAKEPEHPKSHDSKTAPVPVKHQDASKDLKPVKEPEQPKSHDSKSAPIPVKNQEVSKHPE
SVHEKLKEAVKEPEHPKSHDSKTAPVPVKHQDASKDPKQMKEPEQPKSPCSKSAPIPVKNQEVSKHPESVHEKLKEAAKEPEHPKSHDSKTAPVPVK
HQDASKDLKPVKEPEQPKSHDSKSAPIPVKNQEVSKDTKPVHEKPKESLKEPEQPKSHDSKTAPVPVKHQDASKDTKPVKEPEQPKSPCSKSAPIPV
KNQEVSKHPESVHEKLKEAVKEPEHPKSHDSKTAPVPVKHQDASKDLKPVKEPEQPKSHDSKSAPIPVKNQEVSKHPEPVHEKLKEAVKEPEHPKSH
DSKTAPVPVKHQDASKDTKPVKEPEQPKSPCSKSAPIPVKNQEVSKHPESVHEKLKEAVKEPEHPKSHDSKTAPVPVKHQDASKDTKLVKEPEQPKS
HDSKSAPIPVKNQEASKHPESVHEKPKGTVKEPEHPKSHESKSTADPMKHQDASKDTKSVKEPEQPKSPCSKSAPIPVKNQEVSKHPESVHEKLKEA
VKEPEHPKSHDSKTAPVPVKHQDASKDTKLVKEPEQPKSHDSKSAPIPVKNQEASKHPESVHEKPKGTVKEPEHPKSHESKSTADPMKHQDASKDTK
SVKEAEQPKSPCSKSAPIPVKNQEASKHSESVHEKLKEAVKEPEHPKSHDSKTAPVPVKHQDASKDTKLVKEPEQPKSHDIKSAPIQVKNHEASTHP
ESVHEKPKEAVKEPEHPKSHDSKSAADPSEAPRMHPRTQNQ

>Gs_SEDC2
MPHHQSAKSTPVPEKSQEASKDAKQVQETSKHTVKEPAPEHSKNPKPAQEKPQQPIIEPVPDQSKSTSNKSAPIQEKTKEASKDPKPAQEKPQQPIK
EPVPDQSKSTPVSVKQHEDSKNPKPAQEKPQQPIKEPVPDQSKSTSNKSAPIQEKTKEASKDPKPAQEKPQQPIKEPVPDQSKSTPVSVKQHEDSKN
PKPAQEKPQQPIKEPVPDQSKSTSNKSAPIQEKTKEASKDPKPAQEKPQQPIKEPVPDQSKSTPVSVKQHEDSKNPKPAQEKPQQPIIEPVPDQSKS
TSNKSAPIQEKTKEASKDLKPAHEKPQQPIKEPVADQSKSPSNKSEPIQEKTKEASKDPKPAQEKPKESVKEPLPKKPKGSDSNSVHTSQEPSKEPK
PIQEKSKKTSE

>Mu_SEDC1
MPAHHGHPEEHQGASKNPKPVQNPHPVQEEPVKEPEQPKSPCSKSAPVPEKQQEASKNPKPVQHPHPVQDIVKEPVKEPEHPKSPCSKSAPVPEKHQ
EPSKNPKPVQHPHPVQDIAKEPVKEPEHPKSPCSKSAHVPEKQQEASKNPKPVQHPHPVQEKAKEPVKEPEHPKSPCSKSAPVPEKHQEPSKNPKPV
QHPHPVQEKAKEPVKEPEHPKSPCSKSAHVPEKQQEASKNPKPVQEKPKDLVKDPVPEKPKVPCGNPVPTPVHPQEPSKEPKPGQEKSKQPSK

>Mu_SEDC2
MAPHHHPDHHQSKKSAPAPEEPQEASKDQNPVQNPHPVQEKAKEPVKEPEQPKSPCSKSAHVPEKQQEASKNPKPVQHPHPVQEKAKEPVKEPEHPK
SPCSKSAHVPEKHQEPSKNPKPVQDPKPVQDKAKEPVKEPEHPKSPCSKSAHVPEKHHEPSKNPKPVQDPKPVQDKAKEPVKEPEHPKSPCSKSAHV
PEKHQEPSKNPKPVQDPKPVQDKAKEPVKEPEHPKSPCSKSAHVPEKHHEPSKNPKPVQDPKPVQDKAKEPVKEPEHPKSPCSKSAPVPEKHQEPSK
NPKPVQHPHPVQDIAKEPVKEPEHPKSPCSKSAHVPEKQQEASKNPKPVQEKPKDLVKDPVPEKPKVPCGNPVPTPAQEPSKEPKPGQEKSKQPSK

>Mu_SEDC3
MPAHHGHPEEHQGASKNPKPVQNPHPVQEEPVKEPEQPKSPCSKSAPVPEKQQEASKNPKPVQHPHPVQEKAKEPVKEPEHPKSPCSKSAPVPEKHQ
EPSKNPKPVQHPHPVQEKAKEPVKEPEHPKSPCSKSAHVPEKHQEPSKNPKPVQEKPKDLVKVPVPEKPKVPCGNPVPTPAHPQEPSKEPKPGQEKS
KQLSK

>Mu_SEDC4
MSHHQGTKSAPVPEKPQEVPKDPKPMQENPKESEKTPEHPKSPCSKSAPVLEKPQEVSKDPKPVHEKSKEPEKKPELPKSPCSKSAPVLEKPQEASK
DTKPVHEKPKEPEKKPELPKSPCSKSAPVLEKPQEASKDTKPVHEKPKEPEKKPELPKSPCSKSAPILEKPQEVSKDTKPVHEKPKEPEKKPEHPKS
PCSKSAPVLEKPQEVSKDTKPVHEKPKEPEKKPEHPKSPCSKSAPVLEKPQEVSKDTKPVHEKPKEPEKKPEHPKSPCSKSAPVLEKPQEVSKDTKP
VHEKPKEPEKKPELPKSPCSKSAPVLEKPQEVSKDPKPVHEKSKEPEKKPELPKSPCSKSAPVLEKPQEVSKDTKPVHEKPKEPEKKPEHPKSPCSK
SAPVLEKPQEVSKDTKPVHEKPKEPEKKPEHPKSPCSKSAPVLEKPQEVSKDTKPVHEKSKEPEKKPELPKSPCSKSAPVLEKPQETSKDTKPVHEK
PKEPEKKTELPKSPCSKSAPILEKPQEVSKDTKPVHEKPKEPEKKPEHPKSPCSKSAPVLEKPQEVSKDTKPVHEKSKEPEKKPELPKSPCTKSAPV
LEKPHEVSKDPKPVQEKPKEPEKKPENPKSPCSKSAPISEKPHEVTKDPKVVHEKPKEPEKKPEHPKSPCSKSAPVLEKPQEASKDPKPGHEKPKEP
EKKPEHPKSPCSKSAPVLEKPQEASKDPKPVHEKSKEPEKKPELPKSPCSKSAPVLEKPQETSKDTKPVHEKPKEPEKKPELPKSPCSKSAPVLEKP
QETSKDTKPVHEKPKEPEKKPELPKSPCSKSAPVLEKPQEVSKDTKPVHEKPKEPEKKPEHPKSPCSKSAPVLEKPQEASNDPKPGHGKPKEPEKKP
EHPKSPCSKFAPIPEKPHEVSKDPKPVQEKPKEPEKKPEHPKSPCSKSAPVLEKPQEVSKDPKPVHEKPKEPEKKPEHPKSPCSKSAPVLEKPQEAS
KDPKPGHEKPKEPEKKPEHPKSPCSKSAPVLEKPQEASKDPKPGHEKPKEPVKEPMPEKPKGLCGKPFPTPVQPQEPSKEPKPGQEKEPSK

>Rb_SEDC1
MSSHQSTKPVPDKHQEPYKEPKSAPEKPKGTCSSPGPTQGKSQEPSKEPKPVQEKPKEPASQKSKEPSKEPKPVQEKPKEPAQEKSKEPSKEPKQVQ
EKPKEPAKEAKPSPTKPQKP

>Rb_SEDC2
MSNHQSANFAPLPGKPQEQSKEQKLVQEKPKESAKEQAPVKTQEPKEQAPVKTHETKQVQQQPKEQAPVKTQETKHVQEKPKEQAPVKTQETKQVQQ
QPKEQAPVKTQETKEQAPVKTQETKQVQQQPKEQAPVKTQETKQVQQQPKEQAPVKTQETKQVHHQPKEQAPVKTQETKEQAPVKTQETKQVQEKPK
EQAPVKTQETKEQASVKAQETKQVQQQPKEQAPVKTQETKEQAPVKAQETKQVQQQPKEQAPVKTQETKEQAPVKAQETKQVQQQPKEQAPVKTQET
KQVQQQAKEQVPVKTQETKEQAPVKTQETKQVQQQPKVQAPVKTQETKEQAPVKAQETKQVQQQPKEQAPVKTQETKEQAPVKTQETKQVQQQPKEQ
APVKTQETKQVQQQPKEQAPVKIQETKQVQQSPKEQTPVKTQETKEQAPVKTQETKEQAPVKTQETKEQAPVKAQETKQVQEKPKEQAPVKTQETKQ
VQEKPKEQAPVKTQETKEQAPVKAQETKQVQQQPKEQAPVKTQETKEQAPVKAQDTKQVQQQPKEQAPVKTQETKQVQQQAKEQVPVKTQETKEQAQ
VKTQETKQVQQQPKEQAPVKTHETKEQAPVKAQETKQVQQQPKEQAPVKTQETKEQAPVKAQETKQVQQPPKEQAPVKTQETKEQAPVKAQETKQVQ
QQPKEQAPVKTQETKQVQQQAKEQVPVKTQETKEQAPVKTQETKQVQQQPKEQAPVKTQETKKQAPVKSQEIKQVQQQPKEQAPVKTQDTKQVQQQP
KDQAPVKTQETKQVQQQPKEQAPVKTQETKQVQQPKEQAPVKTQETKQVQQQPKEQAPVKSQETKQVQQPKEQAPVKSQETKQVQQQPKEQAPVKTQ
ETKEQAPVKAQETKQVQEKPKEQAPVKTQETKQVQQQPKEQAPVKAQETKEQAPVKTQETKQVQQQPKEQAPVKTQEIKEQAPVKTQETKQVQHQPK
EQAPVKAQETKQVQQQPKEQAPVKTQETKQVQQQPKEQAPVKTQETKEQAPVKTQETKQVQHQPKEQAPVKAQETKQVQQQPKEQAPVKTQETKQVQ
HQPKEQAPVKAQETKQVQQQQPKEQAPVKTQETKQVQEKPKEQAPVKTQEIKQVQQQPKEQAPVKTQETKQVQQQPKEQAPVKTQETKQVHQQPKEQ
APVKTQETKQVQQQPKEQAPVKTQETKQVQQQPKEQAPVKTQETKQVQEKPKEQAPVKTQETKEQVPVKSQETKQVQEKPKEQAPVKSQETKQVQQQ
QPKEQAPVKTQETKQVQQQPKEQAPVKTQETKEQAPVKTQETKQVQHQPKEQAPVKAQEIKQVQQQQPKEQAPVKAQETKQVQQQPKEQAPVKTQET

KQVQHQPKEQAPVKAQETKQVQQQQQPKEQAPVKTQETKQVQEKPKEQAPVKTQETKHVQEKPKEQAPVKTQETKQVQQQPKEQAPVKTQETKQVQQ
QPKEQAPVKTQETKQVQQQPKEQAPVKTQETKQVQEKPKEQAPVKAQETKQVQQQQPKEQAPVKTQETKHVQEKPKEQAPVKSQETKQVQQQQPKEQ
APVKTQETKQVQQQQPKEQAPVKTQETKQVQQQPKEQAPVKTQETKQVQQQPKEQAPVKTQETKEQAPVKSQETKQVQEKPKEQAPVKAQETKQVQQ
QPKEQAPVKAQETKQVQQQPKEQAPVKTQETKQVQQQPKEQAPVKAQEAKQVQQQQPKEQAPVKTQETKQVQQQPKEQAPVKAQETKQVQQQPKEQA
PVKAQETKQVQQQPKEQAPVKTQETKQVQQQPKEQAPVKAQEAKQVQQQQQPKEQAPVKSQETKQVQQQPKEQAPVKAQETKQVQQQQPKEQAPVKA
QETKQVQEKPKEPAKEPAAKKLF

B

>Am_SFTP1
MANLLRALKLIIDTFNSYSRQQGHFDKLNKDEFNLLIQNEFADIIEDSENPQTIAAIMKALDENDDGEVDFKEFMALLFKVALAYYEAEQAEHLKGT
SQVKKQEKQSTTAPHQAPTPKEDPTKVQQKTPAKDDPLKKQDKSLSKQDPPEKQMSHTKEKHEEKEGALSSKKEPLKTQDPPQKQICSSKEKHEEKE
GTLSSKKEPLKTQDPPQKQICSSKEKHEEKEGASVPQKEPQKRRIHPQTK

>Am_SFTP2
MPSFMESIDNITTIFQKYASKECGRDKLNKAELTQLLQDELSPAMKVSGDGSINTILKALDQNGDGQVDFNEFLVLVFKVAKAYNEHVSKSRNLEPC
GVHQPGEPQNKPVAGPRAEPATLPAIKPAPEIETEPDLEDKSIQTPDSRVDTVSNTKPAAHPEATCKISPDTKQAQPGPVLATKPAPVLETKPAPVP
EKSPPTEPKTEPAHVPKTKVVPHPEPTYKVSEKEAPVAPAPGTKPASSPETKPVPVPARSTSSGPKTEPAPVPDPKGVPHPAPMCKAPSDAQTEQAQ
PAPETKAVSAVETPVKPVPEKKATPASVTSKEASPKAATELPTEHAGTWADKTSG

>Am_SFTP3
MPTIVSALQDIVDVFLRYTAQECDRDKLNRAELESIIQNEFTDVIKNPKDPQTPATMLIVLDEDKDGQVDFNEFMNLVCKVAKAYYKTFYPAQGTCD
SKPGQAKDSHNQDQGKQKHPHRQDKQDPPHQDQGKQYGDYQDSARQVQGKNDNSHNETAPHWGTRYDQGKPGGSQAEQGKQDHSYQDHDAKDGSKQD
HGKEKPPHHHHHHGKHSGSHQDEVKKDGSYRDQDKKEGSHQDHGNQYGYHQDQNRKEGSHQDKGMKGDVYHDLDSKNISLPDQANKYGSLLDQGKRD
GSRPDHSNQYGYSHEQGRKEGSNQDQYKKDDSSQTERNQYGHRQDHGSYHDKDNIYGSQNDQANKYGSPQDQGNKYGYHLDQDKKDHSYNVQDSKHG
SYHEQDTNYGSHQDQNKKEDSHQDHSRQYGSQQDQGKQYGSIQDQDKQDTSYQHQGKQDDSQQQQGKQDGSQQQQGKQDGSHRYQEKKESYHHDQGI
EVPSYQYHGMKESSYGDKVNQESHHHHGQDRQGPTHHDDGKQSGSHHDYGKQWSANQDQHKEFGNKQNDNNQGKQEPSHQDKGNKIGLYHGQENQVP
SPQYDEKKVPPPQDQRKQEPSPQYQERKVLPPQDQSKQEPTPLYQESKVPPPQDQSKQEPSPQFQDRKVPPPQDQSKQEPSQQYQEGKVPPPQDQSK
QEPSQQYQESKVPPPQDQSKQEPSTQFQDRMVPPPQDQSKQETSPQYQESKVPPPQDEVKQGLEQQEHSSQVSQQNPSEHHQPQQPHYPQQHSPNRP
VQYHYQYQWSPNQRRFPGQM

>Gs_SFTP1
MPQLLNAIKSVIDTFENYCSKEQGYGKLNKAAFNRLIQSEFADVIEDSENPKTIEAIMQVLDENDDGEVDFREFLDLFFKVIVAYYDALQAYKQKKH
QQPSDSDSKQEKTPQRDSSKKQETPQPQTSILKQEPCMKADILQPKSPSQKQESALLQTPTTASQKKDHSKKEDPLLSSQKQDPSQPKSLSQKPGPF
QPQSPSQKSEAFQKKDLSQPQSPSQKQDSVQSQTPSPNQDLSKKQECSQSHSQSLSQKQNLFPKKESSEKPDSSQSQTLSQPQSHEKKDTSKKQDVS
KKQDLSQPHYSSKTQDPFQHQNPSKKQDQHQPQSPSKKQDPTQIQFPSHQHDPKKEPSKVKEPSIIQKSPLLESTALKEEAFKKQDISQPQSPSKDP
LHPQDSSKKQDPKQILSSSHKLDSCKKQVTSQPQSPSQKEDTSKKQDPLKSQSLSKQHDPLQQKDTSKKEGSTLTLSSSEQQDKSKKQDQSQPHSPS
KTEDPIKHQHPSKKQDQYEPQSPSKKQDQTQHQLPSHQQDPKKEPSKVIEPSILQKSPLLESTALKEEAFKKQDISQPQSPSKDPLHPQDSSKKQDP
KQILSSSHKLDSCKKQVTSQPQSPSQKEDTSKKQDPLKSQSLSKQHDPLQQKDTSKKEGSTLTLSSSEQQDKSKKQDQSQPHSPSKTEDPFKHQHPS
KKQDQYEPQSPSKKQDPTQHQLPSHQQDPKKEPSKVIEPSILQKSPLLESTALKEEAFKKQDISQPQSPSKDPLHPQDSSKKQDPKQILSSSHKLDS
CKKQVTSQPQSPSQKEDTSKKQDPLKSQSLSKQHDPLQQKDTSKKEGSSLTLSSSEQQDKSKKQDQSQPHSPSKTEDPFQHQHPSEKQDPYQPQSPS
KKQDPTQIQSLSHQQDPKKEPSKVIEPSIMQKSPLLESTALKEEAFKKQDISQPQSPSKDPLHPQDSSKKQDPKQILSSSHELDSCKKQVTSQPQSP
SHKEDTSKKQDPLKSQSLSKQHDPLQQKDTSKKEGSTLTLSSSEKQETSKKQDLSQPHSPSKTEDTFQHQHPSKKQDPYQPQSPSKKQDPTQHQFPS
HQQDPKKEPSKVIEPSILQKSPLLESTALKEEAFKKQDISQPQSPSKDPLNPQDSSKKQDPKQILSSSHKLDSCKKQVTSQPQSPSQKEDTSKKQDP
LKSQSLSKQHDPLQQKDTSKKEGSTLTLSSSEKQETSKKQNLSQPHSPSKTEDPFQHQHPSKKQDQYEPQSPSKKQDPTQHQLPSHQQDPKKEPSKV
IEPSILQKSPLLESTALKEEAFKKQDISQPQSPSKDPLHPQDSSKKQDPKQILSSSHKLDSCKKQVTSQPQSPSQKEDTSKKQDPLKSQSLSKQHDP
LQQKDTSKKEGSSLTLSSSEQQDKSKKQDQSQPHSPSKTEDPFQHQHPSKKQDPYQPQSPSKKQDPTQIQFPSHQQDPKKEPSKVIEPSIMQKSPLL
ESTALKEEAFKKQDISQPQSPSKDPLHPQDSSKKQDPKQILSSSHEVDSCKKQVTSQPQSPSHKEDTSKKQDPLKSQSLSKQHDPLQQKDTSKKEGS
TLTLSSSEQQDKSKKQDQSQPHSPSKTEDAFRHQHPSKKQDQYEPQSPSKKQDPTQHQLPSHQQDPKKEPSKVIEPSILQKSPLLESTALKEEAFKK
QDISQPQSPSKDPLHPQDSSKKQDPKQILSSSHKLDSCKKQVTSQPQSPSQKEDTSKKQDPLKSQSLSKQHDPLQQKDTSKKEGSSLTLSSSEQQDK
SKKQDQSQPHSPSKTEDPFQHQHPSKKQDPYQPQSPSKKQDPTQIQFPSHQQDPKKEPSKVIEPSIMQKSPLLESTALKEEAFKKQDISQPQSPSKD
PLHPQDSSKKQDPKQILSSSHKLDSCKKQVTSQLQSPSHKEDTSKKQDPLKSQSLSKQHDLLQQKDTSKKEGSTLTLSSSEQQDKSKKQDQSQPHSP
SKTEDAFRHQHPSKKQDQYEPQSPSKKQDPTQHQLPSHQQDPKKEPSKVIEPSILQKSPLLESTALKEEAFKKQDISQPQSPSKDPLHPQDSSKKQD
PKQILSSSHKLDSCKKQVTSQPQSPSQKEDTSKKQDPLKSQSLSKQHDPLQQKDTSKKEGSSLTLSSSEQQDKSKKQDQSQPHSPSKTEDPFQHQHP
SKKQDPYQPQSPSKKQDPTQIQFPSHQQDPKKEPSKVIEPSIMQKSPLLESTALKKEAFKKQDISQPQSPSKDPLHPQDSSKKQDPKQILSSSHELD
SCKKQVTSQPQSPSHKEDTSKKQDPLKSQSLSKQHDPLQQKKEGSTLTLSSSEKQETSKKEDLSQPHSPSKTEDPFQHQHPSKKQDPYQPQSPSKKQ
DPTQHQLPSHQQDPKKEPSNVKEPSSMQKSPLLESTALKEEDFKKQDTSQPQSPSKDPLHPQDSSKKQDPKQILSSSHKLDSCKKQVTSQPQSPSQK
EDTSKKQDPLKSQSLSKQHDPLQQKDTSKKEGSTLTLSSSEKQETSKKQDLSQPHSPSKTEDPFKHQHPSKKQDQYVPQSPSKKQDPTQHQLPSHQQ
DPKKEPSKVIEPSIMQKSPLLESTALKEEDFKKQDTSQPQSPSKDPLHPQDSSKKQDPKQILSSSHKLDSCKKQVTSQPQSPSQKEDTSKKQDPLKS
QSLSKQHDPLQQKDTSKKEDSSLTLSSSEQQDKSKKQDQSQPHSPSKTEDPFQHQHPSKKQDPYQPQSPSKKQDPTQIQFPSHQQDPKKEPSKVIEP
SIMQKSPLLESTALKEEAFKKQDISQPQSPSKDPLHPQDSSKKQDPKQILSSSHELDSCKKQVTSQPQSPSQKEDTSKKQDPLKSQSLSKQHDPLQQ
KDTSKKEGSTLTLSSSEKQDTSKKQDLSQPHSPSKTEDPFKHQHPSKKQDPYVPQSPSKKQDPTQHQLPSHQQDPKKEPSKVIEPSIMQKSPLLEST
ALKEEAFKKQDISQPQSPSKDPLHPQDSSKKQDPKQILSPSHKLDSCKKQITSQPQSPSQKEDTSKKQDPLKSQSLSKQHDPLQQKDTSKKEGSTLT
LSSSEKQETSKKQDLSQPHSPSKIEDPFQHQHPSKKQDQYEPQSPSKKQDPTQHQLPSHQQDPKKEPSKVIEPSIMQKSPLLESTALKEEDFKKQDT
SQPQSPSKDPLHPQDSSKKQDPKQILSSSHKLDSCKKQVTSQPQSPSQKEDTSKKQDPLKSQSLSKQHDPLQQKDTSKKEDSSLTLSSSEQQDKLKK
QDQSQPHSPSKTEDPFQHQHPSKKQDPYQPQSPSKKQDPTQIQFPSHQQDPKKEPSKVIEPSIMQKSPLLESTALKEEAFKKQDISQPQSPSKDPLH
PQDSSKKQDPKQILSSSHELDSCKKQVTSQPQSPSHKEDTSKKQDPLKSQSLSKQHDPLQQKDTSKKEGSTLTLSSSEKQETSKKQDLSQPHSPSKT
EDPFQHQHPSKKQDPYQPQSPSKKQDPTQHQLPSHQQDPKKEPSNIKEPSSMKKSPLLESTALKEEDFKKQDTSLPQSPSKDPLHPQDSSKKQDPKQ
ILSPSHKLDSCKKQVTSQPQSPSQKEDTSKKQEPLKSQSLSKQHDPLQQKDTSKKEGSILTLSSSEKQETSKKQDLSQPHSPSKIEDPFQHQHPSKK
QDPYEPQSPSKKQDSTQIQFPSHQQDPKKEPSKVIEPSILQKSPLLESTALKEEAFKKQDTSQPKSPSKDSLHPQDSSKKQDPKQILSSSHKLDSCK

KQVTSQPQSPSQKEDTSKKQDPLKSQSLSKPHDPLQQKDTSKKEGSTLSSSEKQETSKKQDLSQPHSPSKTEDPFQHQHPSKKQDPYQPQSPSKKQD
PTQIQFPSHQQDPKKEPSKVIEPSIMQKSPLLESTALKEEAFKKQDISQPQSPSKDPLHPQDSSKKQDPKQILSSSHKLDTCKKQVTSQPQSPSQKE
DTSKKQDPLKSQSLSKPHDPLQQKDTSKKEGSILTLSSSEKQETSKKQDLSQPHSPSKTEDPFQHQHPSKKQDQYEPQSPSKKQDPTQHQLPSHQQD
PKKEPSKVIEPSIMQKSPPLESTALKEEAFKKQDISQPQSPSKDPLHPQDSSKKQDPKQILSSSHKLDSCKKQVTSQPQSPSQKEDTSKKQDPLKSQ
SLSKPHDPLQQKDTSKKEGSILTLSSSEKQETSKKQDLSQPHSPSKTEDPFQHQHPSRKQDPYEPQSPSKKQDSTQIQFPSHQQDPKKEPSKVIEPS
IMQKSPLLESTALKEEAFKKQDTSQPQSPSKDPLHPQDSSKKQDPKQILSSSHKLDTCKKQVTSQPQSPSQKEDTSKKQDPLKSQSLSKPHDPLQQK
DTSKKEGSILTLSSSEKQETSKKQDLSQPHSPSKTEDPFQHQHPSKKQDQYEPQSPSKKQDPTQHQLPSHQQDPKKEPSKVIEPSIMQKSPLLESTA
LKEEAFKKQDISQPQSPSKDPLHPQDSSKKQDPKQILSSSHKLDSCKKQVTSQPQSPSQKEDTSKKQDPLKSQSLSKPHDPLQQKDTSKKEGSILTL
SSSEKQETSKKQDLSQPHSPSKTEDPFQHQHPSKKQDPYEPQSPSKKQDSTQIQFPSHQQDPKKEPSKVIEPSIMQKSPLLESTALKEEAFKKQDTS
QPQSPSKDPLHPQDSSKKQDPKQILSSSYKLDSCKKQVTSQPQSPSQKEDTSKKQDPLKSQSLSKAHDPLQQKDTSKKEGSTLTLSSSEKQETSKKQ
DQSQPHSPSKTEDPFQHQHPSKKQDPYQPQSPSKKQDPTQIQFPSHQQDPKKEPSKVIQPSIMQKSPLLESTALKEEAFKKQDISQPQSPSKDPLHP
QDSSKKQDPKQILSSSHKLDSCKKEVTSQPRSPSHKEDTSKKQDPLKSQSLSKQHDPLQQKDTSKKEGSTLTLSSSEKQETSKKQDLSQPHSPSKTE
DPFQHQHPSKKQDPYQPQSPSKKQDSTQIQFPSHQQDTKKEPSKVEESSLIQKSLFLDSTPSKQDTFPLQSSSQKQDSLHSQSSSLKDLSKNQDLGQ
AQSLSPKQDQSMKDDLVLKPDDPLKKDQVIHERFSEQDTCQKPQDPGQKHDSQSQSQTGIWRQVFPSTHQEKHSSEQAGQSVFYSYLKEFEQKQQSQ
SWGWKHIFPFSQQQDPSQKHDTVREQLAWSSEQHPQHSAAQQAESMFPSQYESPSASVGTPQHSWSQAQGQGQLKKQDPAQPDQRQETPPSHAAPKE
NPSQQQQQPEKQPTKHFRWPWQT

>Gs_SFTP2
MPSLINVIEDTIQVYFKYSQRSCNHQKLNNEELKSLIQNEFADVIKNPNDPQTAETVLKILDENKDGKVDFEEFMDLLAKVAKAYFKTFHKDKSQNT
CQQEQTMSGQQKEIFTEQTDNFRQDTTKQKIIQQDSLEQNMPQPQHPQQISPPKQDYLSHQEPIKSGQDKKDLIKQTSWKDQQESGKTQVDQSQQNS
SNQESWQKPQQNQDTVRRQGLSMEDSPKEYQQKQDPSQQQELNKQTSSQQQWSSQQGSWQEKQYSTDTQGLPHDEKQKHQSQPQQPSYQIDSKQNQT
QDHNKSDLVQERQSQHQQSHHQVHHHDGQKSDLSQQQGFDKQTTSQEQWSSQRSSWQEKEYPIDKQGLSNDEKQKHQSQPQQPSYQIDSKQIQTQDH
NKSDLVQERQSQHQQSHHQVHHHDGQKSDLSQQQGFDKQTTSQQQWSSQQGSWQEKEYPIDKQGLSNDEKQKHQSQPQQPSYQIDSKQNQTQDHNKS
DLVQERQSQHQQSHHQVHHHDGQKSDLSQQQGFDKQTTSQEQWSSQRSSWQEKEYPIDKQGLSNDEKQKHQSQPQQPSYQIDSKQNQTQDHNKSDLV
QERQSQHQQSHHHVHQHDGQKSDLSQKEHQEQEGQHHSQNSSYQLAKDKHDSSREHEHKYSSRQDKYKEDNQEREQKYQSSYPSRQPDKGRQDPSNE
QEGQLYQTQYPPRQQDKYKQDNQEREHDTSHQSDKDRRFPSHEKEEQSYQSQYPPRQQDKYKQDPAQEKEQQYQSQYDQHQQDKNKQGLTEHHSSNL
PQKEQGLGQQEDSGSVEHHDSSQNRHQKIHQRIQYQYQYLWSPYQPKFPWQK

>Mu_SFTP1
MSRLLSAIKSVIDTYDSYCSKEQSHDKLNPEAFNRLIQNEFADVIEDSENPKTIEAIMQVLDENHDGEVDFREFLDLFFKVIVAYYDALQMYKQKKH
QQPLGSDSKQEKTHLQDSSKKQETPQPQTSSLKQEPCKKSDIPQPKPHSQKQESALLQTPPQKQSASKEKDQPKKEDPLLSSQKKDPSQLQSLYQKP
DPLQPQSPSQKPEASQKQDPFLKQDPSHPQSPSQKPETIQKQDPFQKKDPSQPQSPSQKPEISQKQDPFLKKDPTHPQSPPQKPETTQKQDPFLKKD
PSHPQSPSQKPETTQKQDPFLKKDPSHPQSPSQKPDPVQPKSPSQKQEASQKQDPFLKKDPSQPQSPSQKPETTQKQDPFQKKDPSQPQSPSQKLDP
IEKQGKSQKQESYQKKETPLVESTPLKQDLSQKKDPSHPQSPSQKPETSQKQDPFQKKDPSQQQSLSQKPETSQTQDPFQKKDPSHPQSPSQKPETS
QKQDPFQKKDPSHPQSPSQKPEPSQKQDPFQKKDPSHPQSPSQKLDPIEKQGMSQKQESHQKKETSLVESSPLKQDLSQKHDPSHPQSPSQKPETIQ
KQDPFLKKDPSHPQSPSQKPETTQKQDPFQKKDPSHPQSPSQKPEASQKQDPFQKKDPSHPQSPSQKPETSQKQDPFQKKDPSLPQSPSQKLDPIEK
QGMSQKQESHQKKETSLVESSPLKQDLSQKHDPSHPQSLSQKSEVSQKQDPFLKKDPSHPQSPSQKPETTQNQDPFQKKDPSHPQSPSQKPETSQKQ
DPFLKKDPSHPQSPSQKPEASQKQDPFQKKDPSQPQSPSQKPETTQKQDPFQKKDPSQPQSPSQKLDPIEKQGMSQKQESHQKKETSLVESSPLKQD
LSQKHDPSHPQYLSQKPETTQKQDPFQKKDPSQPQSPSLKPETSQKQDPFLKKDPSHPQSPSQKPETSQKQDPFQKKDPSQPQSPSQKLDPIEKQGM
SQKQESYQKKETPLVESSPLKQDLSQKQDPSHPQSLSQKPETSQKQDPFHKKDPSHPQSPSQKPETSQKQDPFQKKDPSQPQSPSQKPETSQTQDPF
QKKDPSHPQSPSQKPETSQKQDPFLKKDPSHPQSPSQKPEASHKQDPFQKKDPSHPQSPTQKLDPIEKQGMSQKQESYQKKETPLVESSPLKQDLSQ
KQDPSHPQSLSQKPETSQKQDPFQKKDPSQPQSPSQKPETSQTQDPFQKKDPSHPQSPSQKPETSQKQDPSLKKDPSHPQSPSQKPEASHKQDPFQK
KDPSQPQSPSQKLDPIEKQGMSQKQESYQKKETPLVESSPLKQDLSQKQDPSHPQSLSQKPETSQKQDPFHKKDPSHPQSPSQKPETSQKQDPFQKK
DPSQPQSPSQKPETSQTQDPFQKKDPSHPQSPSLKPETSQKQDPFLKKDPSHPQSPSQKPEASHKQDPFQKKDPSHPQSPSQKLDPIEKQGMSQKQE
SYQKKETPLVESSPLKQDLSQKQDPSHPQSLSQKPETSQKQDPFQKKDPSQPQSPSQKPETTQKQDPFQKKDPSQPQSPSQKLDPIEKQGMSQKQES
HQKKETSLVESSPLKQDLSQKHDPSHPQYLSQKPETTQKQDPFQKKDPSQPQSPSLKPETSQKQDPFLKKDPSHPQSPSQKPETTQKQDPFQKKDPS
QPQSPSQKLDPIEKQGMSQKQESYQKKETPLVESSPLKQDLSQKQDPSHPQSLSQKPETSQKQDPFHKKDPSHPQSPSQKPETSQKQDPFQKKDPSQ
PQSPSQKPETSQTQDPFQKKDPSHPQSPSQKPETSQKQDPFLKKDPSHPQSPSQKPEASHKQDPFQKKDPSHPQSPTQKLDPIEKQGMSQKQESYQK
KETPLVESSPLKQDLSQKQDPSHPQSLSQKPETSQKQDPFQKKDPSQPQSPSQKPETSQTQDPFQKKDPSHPQSPSQKPETSQKQDPSLKKDPSHPQ
SPSQKPEASHKQDPFQKKDPSQPQSPSQKLDPIEKQGMSQKQESYQKKETPLVESSPLKQDLSQKQDPSHPQSLSQKPETSQKQDPFHKKDPSHPQS
PSQKPETSQKQDPFQKKDPSQPQSPSQKPETSQTQDPFQKKDPSHPQSPSLKPETSQKQDPFLKKDPSHPQSPSQKPEASHKQDPFQKKDPSHPQSP
SQKLDPIEKQGMSQKQESYQKKETPLVESSPLKQDLSQKQDPSHPQSLSQKPETSQKQDPFHKKDPSHPQSPSQKPETSQKQDPFQKKDPSHPQSPS
QKPETSQKQDPFLKKDPSHPQSPSQKPEASHKQDPFQKKDPSHPQSPSQKLDPIENKGMSQKQESYQKKETPLVESSPLKQDLSQKQDPSHPQSLSQ
KPETSQKQDPFHKKDPSHPQSPSQKPETSQKQDPFQKKDPSHPQSPSQKPETSQTQDPFQKKDPSHPQSPSQKPETSQIQDPFLKKDPAHPQSPSQK
PEASHKQDPFQKKDPSHPQSPSQKLDPIEKQGMSQKQESYQKKETPLVESAPLKQDLSQKQDPSHPQSLSQKPETSQKQDPFHKKDPSHPQSPSQKP
ETAQKQDPFQKKDPSHPQSPSQKPETSQTQDPFLKKDPAHPQSPSQKPEASHKQDPFQKKDPSHPQSPSQKLDPIEKQGMSQKQESYQKKETSLVES
SPLKQDLSQKQDPSHPQSLSQKPETSQKQDPYQKKDPSHPQSPSQKPEICQKQDPFQKKDPSHPQSPSQKPDPVQPKSPSQKPEASQKQDPFLKKDL
SHPHPPSQKPETSQKQDPFQKKDPSQPQSPSQKLDPIGKQGMSQKQESHQKKETPLVESSPLKQDLSQKQDPSHPQYLSQKPETSQKQDSFQKKDPS
QPQSPSQKPETSQKQDPFLKKDPSHPQSPSQKPETTQKQDPFQKKDPSQPQSPSQKLDPIGKQGMSQKQESHQKKETPLVESSPQKQDLSQKQDPSH
PQSMSQKPEASQKQDPFLKKDPSHPQSPSQKPETSQKQDPFLKKDPSQPHSPSQKPETAQKQDPFQKKDPSQPQSPSQKPETSQTQDPFLKKDPSHP
QSPSQKPETSQTQVPFQKKDPSHPQSPSQKLDPIGKQGMSQKQESHQKKETPLVESSPLKQDLSQKQGPPHPQSLSQKPETTQKQDPFQKKDPSQPQ
SPSQKPETSQTQDPFQKKDPSQPQFPSQKLDPIGKQGMSQKQESHQKKETPLVESSPLKQDLSQKHDPSHPQSPSQKPETSQKQDPSQKPQSPSKKQ
DPSQHQLPSHQQDPKKEPSKEKDPSLIQKSPLLQSTPQKQDASKKQDIPQPQSPSKDLFQQQDTTKKQETTQKMSSSQKQDTSKKQDTTQPQTRSQK
EETSTKQDPSQQKLTSKNQDPFQQQDTSKKQDPTQPQTASQKQDTPKQQDPKQQLSPSQTQNTKKQDTLQPKSSSKSQDPFHQQDTSKKEDTKEPLS
PSQKQDTSRKQDPSQPKSPSKISDPFQQPCSTKKQDPSQQQSPSHEKDPKKEPSKEKEPSLIQNHHFYSLLPRKKMILRSKIHLSHNHHLKILSSTK
IHLRNKIQYNHCFLLKNRIHQRNKIHHRNTLLLKKKIHQRNKIHHNQSPHPKLKILSSNHAQLKNKIHINHHLLPKNKTHPSISCLPINKMLRKNHQ
RKKNQPRYKNHLFYSLLHRNKMLLRNKIHLSHNPHPKIIFSNRTHLRNKIQNTHCLLLKYKIPPRNKIQHSHSPHPKLKILSSNRVHVRNKIHLNHN
PLPKNKTHPSISCLPINKMLRKNQPRYKNHHFYSLLHRNKMLLRNKIYLSHNPHPKIISSNRTHLRNKIQNTHCLPLKYKIPPRNKIQHSHSPHPKL
KILSSNRVHVRNKIHLNHNPLPKNKTHPSISCLPINKMLRKNHQRKKNQPRYKNHLFYSLLHRNKMLLRNKIHLSHNPHPKIISSNRTHLRNKIQNT
HCLLLKYKIPPRNKIQHSHSPHPKLKILSSNRVHVRNKIHLNHNPLPKNKTHPSISCLPINKMLRKNHP

>Mu_SFTP2

MPFLISAIQDIIEVYLSYAHRECNLQKLNKEELKSLIQNEFADVIKNPNDPQTAVTVMKTLDDNRDGEVDFEEFMDLMSKVSKAYYKTFHKDQSQNT
CRQQPTKPGQQKEPFTEQKDHFRQDTTPQKPTQQGECKQTDSLEQNVPQHQYKDDTPSKQDYLHQQDPIKPGQDKKDPIQQTPWKDQQGSNKTQVDQ
SQQSSSHQESWQKNQQKQDPVRQQGLSNEDSPKEYQQKQDPSQQQGFDKQATSQQKWSSQKDSWQEKHPTDNDEKHQQQPQPQHPSYQKDSKQNQTQ
DNNNSNLAQERQTQHQQSQYSVRQQEGQKSDLSQQQGIDNQATSQQKWSSQKDSWQEKHPTDNDEKHQQQPQPQHPSYQKDSKQNQTQDNNNSNLAQ
ERQTQHQQSQYSVRQQEGQKSDLSQQQGIDNQATSQQKWSSQKDSWQEKYPTDKSGLPNDEKLKQQHQPQHPSYQKDNQTQDNNKSDLAQERQPQHQ
QPQYPVRQQDGQKSDLSQQQGFDKQTTSQQQQSSQQQWSSQQCSWQEKKYSNDEQDLSNDEKQQQQHQPQHPSYQKDSKQNQTQDNNNSNLAQERQP
QHQQPQYPVRQQDGQKSDLSQQQGFDKQTTSQQQQSSQQQWSSQQCSWQEKKYSNDEQDLSNDEKQQQQHQPQHPSYQKIANKIKLKTITTQI

>Rb_SFTP1
MPQLLTIIKDIINTFYSYCNKEDLHGKLNKEAFNLLIQNEFADIIEDSEKPETIAAIMQALDENNDGEVDFKEFLDLFFKVAVAYCEALALYKQKKQ
EKCQQSSGSDSKQQKSSEQDSSKKHETEPKTTPKQDPSLQQTPPQKQDTSHPQPFSQEKDPSQKQKPAQSKSSSQSLSQKPETSQKQDPSQPQFSYQ
KHDSIQKQGMSQKQDSTQKQEPCQTKNKPLVESSPQKQDAPQLKSPSHKQDPLQKKDISEKHDLSKPQSLSQQHDLSKKPELSQPKCPAKKIDPFQQ
QSPTQKQDPSQPQSPTQKQDISKKPEPAQPKSPSKKIDPFQQQSPNQKQDPSQPQSPTQKQDISKKSEPALAKSPFKKQDISTAKEPAKKQDASQMQ
ELPLLKSSPPKQDASKQQDPSQPQALSQKEDPCKPHDSSQPLSPSTKQGSSKKQDPSLPLFPSKKEDPSQPQSHSQKQDPFQKKDPSKKAEPLKIQD
QSFTQESPLLQSLPQKQDPSQPQAPSQKQEPIQEQDQSKKQDPSKKQSPSQKLESLKKQDPSKKKDPSQPQSPSQKQDPVQKQDPLKKKEPSQPQSP
SKKQDQIQKQDSSQQQSPSTKDSCKKQDPPKKGQSLKKHDETLTQESPLLQSLPQKQDPSQTQFPSKKQDISKKEELSKPQSPSPKQDHFQKQDPLK
KQEPSQPKSDSQKQESIHKEDTSKKQDPFQPQSPSQKQDTFQKEDLSKPQSPSQKQDHVQKQDPVKKQEPSQPKSPSQKQESIHKEDPSKKQEPSKP
ESPSQKQEPFQKQDSPQSHSPSQKQDHFQKQDPLKKQDPSQTQSPSLKQDPIQQQDPIEKQDSSQQQSPSTKDSCQKQDPSKKGQPLKKHDQSQTQE
SPLLQSLPQKQDISKKEELSQPQSPSQKQDHLQKQDPLKKQEPSQPKSPSQKQESIHKEDPSKKQDPFQPQSPSQKQDTFQQQDPLKKHPCQPQSPS
QKQDHFQKEDLSKPQSPSQKQDHFQKQDPLKKQEPSQPKSPSQKQESIHKEDPSKKQDPFQPQSPSQKQDTFQQQDPIKKHPCQPQSPSQKQEHFQK
EDLSQPQSPSQKQDHFHKQDPLKKQESSQPKSPSQKQESIHKEDPSKKQDPFQPHSPSQKQDTLQQQDPLKKQPCQPQSPSQKQDHFQKEDLSKPQS
PSQNKDHFQKEDPLKKQEPSQPKSPSQKQESIHKEDPSKKQDPFQPHSPSQKQDTLQQQDPLKKHPCQPQSPSQKQEHFQKEDQSQPQSPSQKQDHL
QKQDPLKKQEPSQPKSPSQKQESIHKEDPSKKQDPFQPHSPSQKQDTLQEQDPVKKHPCQPQSPSQKQEHFQKEDLSQPQSPSQKQDHFQKQDPLKK
QESSQPKSPSQKQESIHKEDPSKKQDPFQPHSPSQKQDTLQQQDPLKKHPCQPQSPSQKQDHFQKEDLSKPQSPSQKQDHFQKQDPLKKQEPSQPKS
PSQKQESIHKEDPSKKQDPFQPHSPSQKQDTLQQQDPLKKHPCQPQSPSQKQDHFQKEDLSKPQSPSQKQDHFQKQDPLKKQEPSQPKSPSQKQESI
HKEDPSKKQDPFQPQSPSQKQDTLQQQDPLKKHPCQPQSPSQKQEHFQKEDLSKPQSPSQKQDHLQKQDPLKKQEPSQPKSPSQKQESIHKEDPSKK
QDPFQPQSPSQKQDTFHQQDPLKKHPCQPQSPSQKQDHFQKQDPLKKQEPSQPKSPSQKQESIHKEDPSKKQDPFQPQSPSQKQDTFQQQDPLKKHP
CQPQAPSQKQEHFQKEDLSQPQSPSQKQDHFHKQDPLKKQESSQPKSPSQKQESIHKEDPSKKQDPFQPHSPSQKQDTLQQQDPLKKHPCQPQSPSQ
KQEHFQKEDLSKPQSPSQKQDHLQKQDPLKKQEPSQPKSPSQKQESIHKEDPSKKQDPFQPQSPSQKQDTFHQQDPLKKHPCQPQSPSQKQDHFQKQ
DPLKKQEPSQPKSPSQKQESIHKEDPSKKQDPFQPQSPSQKQDTFQQQDPLKKHPCQPQAPSQKQEHFQKEDLSQPQSPSQKQDHFHKQDPLKKQES
SQPKSPSQKQESIHKEDPSKKQDPFQPHSPSQKQDTLQQQDPLKKHPCQPQSPSQKQEHFQKEDQSQPQSPSQKQDHLQKQDPLKKQEPSQPKSPSQ
KQESIHKEDPSKKQDPFQPQSPSQKQDTLQQQDPLKKHPCQPQSPSQKQEHFQKEDLSKPQSPSQKQDHLQKQDPLKKQEPSQPKSPSQKQESIHKE
DPSKKQDPFQPQSPSQKQDTFHQQDPLKKHPCQPQSPSQKQDHFHKQDPLKKQESSQTKIPFPKARIYP

>Rb_SFTP2
MPSIIGAIQDLIDVFHSYSKEQCKLLNKDELKSLLQKELANVIKNPNDPQTVETVMKILDDNQDGGVDFNEFTDLMCKIIKAYYNTFHMEQSQDTCQ
QQQTKPGQQNQPFTEQKDQSKKESTNQKSTQQDKCEQGPYIKADQLEQKPPQHQYKHEQPQQLVPSKEVPSQPDPSYHQGKYESPKQVVPPKQDPSQ
SDSSYHQGKNEPPKQVVPPKQDPSQPDASYHQGKYEPPQQLVPPKQDPSQSDSSYHQGKYEPPKQVVPPKQDPSQSDPSYHQGKYEPPQQLVPPKQD
PSQSDSSYHQGKYEPPQQLVPPKQDPSQSDSSYHQGKYEPPKQVVPPKQDPSQSDPSYHQGKYEPPQQLVPPKQDPSQSDSSYHQGKYEPPQQLVPP
KQDPSQSDSSYHQGKYEPLKQVVPPKQDPSQSDPSYHQGKYEPPQQLVPPKQDPSQSDSSYHQGKYEPPKQVVPPKQDPSQSDPSYHQGKYEPPQQL
VPPKQDPSQSDSSYHQGKYEPPKQVVPPKQDPSQSDPSYHQGKYEPPQQLVPPKQDPSQSDSSYHQGKYEPPQQLVPPKQDPSQSDSSYHQGKYEPP
KQVVPPKQDPSQSDPSYHQGKYEPPQQLVPPKQDPSQSDSSYHQGKYEPPQQLVPPKQDPSQSDSSYHQGKYEPPKQVVPPKQDPSQSDPSYHQGKY
EPPQQLVPPKQDPSQSDSSYHQGKYEPPKQVVPPKQDPSQSDPSYHQGKYEPPQQLVPPKQDPSQSDSSYHQGKYEPPKQVVPPKQDPSQSDPSYHQ
GKYEPPQQLVPPKQDPSQSDSSYHQGKYEPPQQLVPPKQDPSQSDSSYHQGKYEPPKQVVPPKQDPSQSDPSYHQGKYEPPQQLVPPKQDHLQQQES
IKSGQDNKDPPQQTPWKNQEELDKTQQSPGQQDPSKEEEQHSYSKYEYHLRKQTDHTHDPPQEEPHPVAQHVPSQHRQQQVHYQYQYLWSPNQCRFP
WQK

>Xt_SFTP1
MAKLLAAVRGTIEAFNLYRSQSSSCNSLSPEEFKRLIEKEFVDIIKESKQPETIKKVLQSLDTKSQKEVNFKDFMEIFKNVSVAYYDASCQLPKEKE
MIVKEHKEQLLPKIPPEDPMDPKGKTKKDDHAQQSEPKQKPVTPVKDDHPHKDEPIKKDDQAQKSDPKQKPAAPVKDDHPHKDEPIKKDNQAQKSDP
KKKPAAPVKDDHPHKDEPIKKDDQAQKSDPKKKPAAPVKDDHPHKDEPIKKDDQAKKSDPQQKPAAPVKDDHPHKDEPIKKDDQAQKSDPKKKPAAP
VKDDHPHKDEPIKKDDQAKKSDPQQKPAAPVKDDHPHKDEQIKKDNQAQKSDPQQKPAAPVKDDHPHKDEPIKKDDQAQKSDPKQKPAAPVKDDHPH
KDHPIKKDDQAQKSDSKQKPAAPVKDDHPHKDHPIKKDDQAQKSDSKQKPAAPVKDDHPHKDEPIKKDDQAQKSDPKQKPAAPVKDDHPHIDEPVKK
DHQAQKSDPKKKPAAPVKDDHPHKDEPIKKDDQAQKSDPKQKPAAPVKDDHPHKDEPIKKDDQAQKSDPQKKPAAPVKDDHPHKDELIKKDDQAQKS
DPKKKPAAPVKDDHPHKDEPIKKDDQAQKSDPKQKPAAPVKDDHPHKDEPIKKDNQAQKSDPQQKPAAPVKDDHPHKDEPIKKDDQAHKSDPKQKPA
APVKDDHPHKDEPIKKDDQAKKSDPKQKPAAPVKDDHPHKDEPIKKDDQAKKSDPQQKPAAPVKDDHPHKDEPIKKDDQAQKSDPKKKPAAPVKDDH
PHKDEPIKKDDQAQKSDPKQKPAAPVKDDHPHKDEPIKKDDQAQKSDPQQKPAAPVKDDHPHKDDPIKKDDQAQKSDPKQKPAAPVKVDTPHKDEPI
KKDDQAKKSDPQQKPAAPVKEEQPHKDEPIKKDDQAQKSDPQKKPAAPVKHDHPPKDEPIKKDDYAQKSDPKKKPAAPVKEGHCYKDHPIKKEDQAQ
KSDSKQKPAAPVKDDHPHKDEPIKKDDQAKKSDPQQKPAAPVKDDHPHKDDPIKKDDQAQKSDPKQKPAAPVKDDHPHKDDPIKKDDQAQKSDPKQK
PAAPVKDDHPHKDEPIKKEDQAQKSDPKQKPAAPVKDDHPHKDEPIKKDDQAQKLDPKKKPAAPVKDDHPHKDEPIKKEDQAHKSDPKQKPAAPVKD
DHPHKDDPIKKDDQAQKSDPKQKPAAPVKDDHPHKDEPIKKEDQAQKSDPKQKPAAPVKDDHPHKDEPIKKDDQAQKLDPKKKPAAPVKDDHPHKDE
PIKKEDQAHKSDPKQKPAAPVKDDHPHKDEPIKKDDQAKKSDPQQKPAAPVKDDHPHKDDPIKKDDQAQKSEPKKKPDTPVKVDIPHKDEPIKKYDQ
AKKSDPQQKPVAPVKDDHPHKHEPIKKDDQAQKLDPKKKPAAPVKDDHPHKDEPIKKEDQAQKSDPKKKPAAPVKEGHCYKDHPIKKEDQAQKSDSK
QKPAAPVKDDHPHKDEPIKKDDQAKKSDPQQKPAAPVKDDHPHKDEPIKKDDQAQKSDPKQKPAAPVKDDHPHKDDAIKKDDQAQKSDPKQKPAAPV
KDDHPHKDDPIKKDDQAQKSDPKQKPAAPVKDDHPHKDDPIKKDDQAQKSDPKQKPAAPVKDDHPHKDEPIKKDDQAQKSDPKQKPAAPVKDDHPHK
DEPIKKDDQAQKSDPKQKPAAPVKDDHPHKDEPIKKDDQAQKLDPKKKPAAPVKDDHPHKDEPIKKEDQAQKSDPKQKPAAPVKEGHCYKDHPIKKE
DQAQKSDSQQKPAAPVKDDHPHKDEPIKKDDQAKKSDPQQKPGAPVKVDIPHKDEPIKKDDQAKKSDPQQKPVAPVKDDHPHKDEPTKKDDQAKKSD
PKQKPVAPVKDDHPHKDEPIKKDDQPQKSDPKQKPAAPVKDDHPHKDEPIKKDDQAKKSDPQQKPAAPVKVDIPHKDEPIKKDDQAQKSDPQQKPAA
PVKDDHPHKDEPIKTDDQAQKSDPKQKPAQPVKDDHPHKDEPIKKDDQAQKSDPKQKPAPPVKDDHPHKDEPIKKDEQAQKSDPKQKPVTPVKDDHP
QKVEPIKKDDYAQKSDPKQKPAAPVKVEIPHKDEPIKKDNQAQKSDPKQKPAAPVKVEIPHKDEPIKKDDQAQKSDPKQKPAAPVKDDHPHKDEPIK
KDDQAQKSDPKQKPAAPVKEDHPHKDEPIKKDDQAQKSDPKQKPAAPVKVEIPHKDEPIKKDDQAQKSDPKQKPAAPVKDDHPHKDEPIKKDDQAQK
SDPKQKPAAPVKEDHPHKDEPIKKDDQAQKSDPKQKPAAPVKDDHPHKDEPIKKDDQAQKSDPQQKPAAPVNVDIPHKDEPIKKDDQAQKSDPQQKP
AAPVKDYHPHKDEPIKKDDQPQKSDPKKKPAAPVKEGHCYKDEPIKKEDQAQKSDSKQKPAAPVKDDHPHKDEPIKKDDQAKKSDPQQKPAAPVKDD

HPHKDEPIKKDDQAKKSDPQQKPAAPVKDDHPHKDEPIKKDDQAKKSDPQQKPAAPVKDDHPHKDEPIKKDDQAQKSDPKQKPAAPVKDDHPHKDEP
IKKDDQAQKSDPQQKPAAPVKDDHPHKDEPIKKDDQAKKSDPQQKPAAPVKDDHPNKDEPIKKDDQAKKSDPKQKPAAPVKDDHPHKDEPIKKDDQA
QKSDPKQKPAAPVKEDIPHKDEPIKKDDHVQKSDPNQKPAAPVKDDHPHKDEPIKKDDHVQKSDPNQKPAAPVKEGHCHEKDYYDASCDLKVLKKLI
FDEYPFPDPKQDPIKTYDNTQKSDPKKKPATPVKDDPPHKDEPIKKDDQSQKSDPKQKPAEPVKDDPPHKDEPIKKDDQAQKSDPKQKPAAPVKDDH
PHKDEPIKKDDHQQKSDPKQKPAAPVKDDHPHKDEPIKKDDQAQKSDPQQKPAAPVKDDHPHKVEPIKKDDQAQKSDPKQKPAAPVKDDHPHKDEPI
KKDDQAQKSDPKQKPAAPVKDDHPHKDEPIKKDDQAQKSDPKQKPAAPVKDDHPHKDEPIKKDDQAQKSDPKQKPAAPVKVEIPHKDEPIKKDDQAQ
KSDPKQKPVAPVKDDHPHKDEPIKKDDQAQKSDPKQKPAAPVKDDHPHKDDPIKKDDQAQKSDPKQKPAAPVKDDHPHKDEPIKKDDQAQKSDPKQK
PAAPVKDDHPHKDEPIKKDDQAQKSDPKQKPAAPVKDDHPHKDDPIKKDDQAQKSDPKQKPAAPVKDDHPHKDEPIKKDDKAQKSDPKQKPAAPVKD
DHPHKDEPIKKDDQAQKSDPKQKPAAPVKDDHPHKDEPIKKDDQAQKSDPKQKPGAPVKVDIPHKDEPIKKDDQAQKSDPKQKPAAPVKEGHCYKDH
PIKKEDQAQKSDPKQKPAAPVKDDPPHKDEPIKKDDQAQKSDPKQKPAAPVKDDHAHKEEPIKKDDQAQKSDPKQKPAAPVKVDIPHKDEPIKKDDQ
AQKSDPKQKPAAPVKDDHPQKDEPIKKDDQAQKSDPKQKPTAPVKEDHPHKDEPIKKDDQAQISEPKKKPDSPVKDDHPQKDEPIKKDDQAQKSGPK
QKPAAPVKVDIPHKEEPIKKDDQAQKSDPKQKPAAPVKDDHPQKDEPIKKDDQAQKSDPKQKPTAPVKEDHPHKDEPIKKDDQAQISEPKKKPDSPV
KDDHPQKDEPIKKDDQAQKSDPKQKPAAPVKDDHPQKDEPIKKDDQAQKSDPKQKPAAPVKEDHPHKDEPIKKDDQAQISEPKKKPDTPVQVDIPHK
DKPIKKDDHAQISEPKKKPDSPVKVDIPHKDEPIKKDDHAQVSEPKKKPDTPVQVDIPHKDEPIKKDDHAQVSEPKKKPDSPVKVDIPHKDEPIKKD
DHAQVSEPKKKPDTPVKVDIPHKDDPIKKDDHAQISEPKKKPAAPVKDDHPHKDEPIKKDDHQQKSDPKQKPAAPVKDDHTHKDEPIKKDDQAQKSD
PKQKPAAPVKDDHPHKDEPIKKDDQAQKFDPKQEPAAPVKDDHPHKDELIKKDDQAQKSDPKQKPAAPVKDDHPHKDEPIKKDDHAQISEPKKKPDT
PVKVDIPHKDKPIKKDDHAQISEPKKKTDSPVKVDIPHKDDPIKKDDHAQISEPKKKPDTPVKVDIPHKDEPIKKDDHAQKSDHKQKPVAPVKDDHP
HKDEPIKKDDQAPKSDPQQKPAAPVKDDHPHKDDPIKKDDHDSIHKQDESTKPKHSVIPDIKTKEVSTKKDDETQTAELTLDPSKNQCQTTKHDPKE
ELSSQSQCPSLLVSKHGAYSIEEEEDEADSSDPLTYYTYFETKYTQYHGQVLSHNVAQQGPSAMPSSPYYEYSLQTPCREFFQTSEYKYFESSQKTS
EKPSSDAQKSLAGAQGGPPSGSSEEKQEKPKSPYKFKWPWH

>Xt_SFTP2
MPKLTGVINEIIGIFLSYSEGTCAPHKLNKNQMNRLIQNEFSDVIKNPKDPKTVEALIKVLDQNKDEEVDFDEFSDLLCKVLKAIYRAMQQMEKDCP
KKAPVHKTQEETDKKPHDVHVEHHDHPDSPSKYYQEPVYPILDKTKEQKDQGKILTSHVQSTDERSKLTTLQTGQTATTTVTTDPVKPVTQITQDKK
DQVLKQDRTVTQIQQDKDKKDQVPYLKKIDTHIHHDKKDQVTQLDIPVTQIQQDKDKKDQVTQLDKTVTQIQQDKDKKDQVPYLKKIDTHIHHDKKD
QVTQLDLPVTQIQQDKDKKDQVTQLDKTVTQIQQDKDKKDQVTQLDKTVTQIQQDKDKKDQVTQLDKTVTQIQQDKDKKDQVTQLDKTVTQIQQDKD
KKDEVPYLKKIDTHIHHDKKDQVTQLDIPVTQIQQDKDKKDQVTQLDKTVTQIQQDKDKKDEVPYLKKIDTHIHHDKKDQVTQLDIPVTQIHHDKDK
KDQVTQLDKTVTQIQQDKDKKDQVPYLKKIDTHIHHDKKDQVSQLDKTVTQIQQDKDKKDQVTQLDKTVTQIQQDKDKKDQVTQLDKTVTHVQQDKK
DQVTQQGISTTHVVQQPGISTTHVAQQPGISTTQVVQRPGISTTQVAPQPRISTTQVVTQPGISTTHVVQQPGISTTQVAPQPRISTTQVVTQPGIS
TTHVVQQPGISTTQVATQPGISTTQVATQPGISTTQVVQRPGISTTQVVQRPGISTTQVTQQGPRSTQTPQTMPSATQTKPCIDQQPPSMYQEHQIP
EMPQMYQQAQQHVHQYQSHFTTQKKKP

Figure S1. Amino acid sequences of proteins encoded by EDC genes of amphibians. **(A)** Amino acid sequences of proteins encoded by SEDC genes of caecilians. **(B)** Amino acid sequences of SFTP genes of amphibians. The following amino acid residues are highlighted to indicate residues important for protein cross-linking and the peculiar amino acid compositions: lysine (K) and glutamine (Q) are potential sites of transglutamination and proline (P) is the residue with the strong potential to disrupt alpha-helix and beta-sheet structures. SEDC, Simple epidermal differentiation complex gene; SFTP, S100 fused-type protein. Am, *Ambystoma mexicanum*; Gs, *Geotrypetes seraphini*; Mu, *Microcaecilia unicolor*; Rb, *Rhinatrema bivittatum*; Xt, *Xenopus tropicalis*.

```
MSNHQS---ANFAPLPGKP-
QEQSKEQKLVQEKPKESAKE
QAPVKTQEPKEQAPVKTHET
KQVQQQ--PKEQAPVKTQET
KHVQE--KPKEQAPVKTQET
KQVQQQ--PKEQAPVKTQET
---------KEQAPVKTQET
KQVQQQ--PKEQAPVKTQET
KQVQQQ--PKEQAPVKTQET
KQVHHQ--PKEQAPVKTQET
---------KEQAPVKTQET
KQVQE--KPKEQAPVKTQET
---------KEQASVKAQET
KQVQQQ--PKEQAPVKTQET
---------KEQAPVKAQET
KQVQQQ--PKEQAPVKTQET
---------KEQAPVKAQET
KQVQQQ--PKEQAPVKTQET
KQVQQQ--AKEQVPVKTQET
---------KEQAPVKTQET
KQVQQQ--PKVQAPVKTQET
---------KEQAPVKAQET
KQVQQQ--PKEQAPVKTQET
---------KEQAPVKTQET
KQVQQQ--PKEQAPVKTQET
KQVQQQ--PKEQAPVKIQET
KQVQQS--PKEQTPVKTQET
---------KEQAPVKTQET
---------KEQAPVKTQET
---------KEQAPVKAQET
KQVQEK--PKEQAPVKTQET
KQVQEK--PKEQAPVKTQET
---------KEQAPVKAQET
KQVQQQ--PKEQAPVKTQET
---------KEQAPVKAQDT
KQVQQQ--PKEQAPVKTQET
KQVQQQ--AKEQVPVKTQET
---------KEQAQVKTQET
KQVQQQ--PKEQAPVKTHET
---------KEQAPVKAQET
KQVQQQ--PKEQAPVKTQET
---------KEQAPVKAQET
KQVQQ--PPKEQAPVKTQET
---------KEQAPVKAQET
KQVQQQ--PKEQAPVKTQET
KQVQQQ--AKEQVPVKTQET
---------KEQAPVKTQET
KQVQQQ--PKEQAPVKTQET
---------KKQAPVKSQEI
KQVQQQ--PKEQAPVKTQDT
KQVQQQ--PKDQAPVKTQET
KQVQQQ--PKEQAPVKTQET
KQVQQ---PKEQAPVKTQET
KQVQQQ--PKEQAPVKSQET
KQVQQ---PKEQAPVKSQET
KQVQQQ--PKEQAPVKTQET
---------KEQAPVKAQET
```

```
KQVQE--KPKEQAPVKTQET
KQVQQQ--PKEQAPVKAQET
---------KEQAPVKTQET
KQVQQQ--PKEQAPVKTQEI
---------KEQAPVKTQET
KQVQHQ--PKEQAPVKAQET
KQVQQQ--PKEQAPVKTQET
KQVQQQ--PKEQAPVKTQET
---------KEQAPVKTQET
KQVQHQ--PKEQAPVKAQET
KQVQQQ--PKEQAPVKTQET
KQVQHQ--PKEQAPVKAQET
KQVQQQQ-PKEQAPVKTQET
KQVQEK--PKEQAPVKTQEI
KQVQQQ--PKEQAPVKTQET
KQVQQQ--PKEQAPVKTQET
KQVHQQ--PKEQAPVKTQET
KQVQQQ--PKEQAPVKTQET
KQVQQQ--PKEQAPVKTQET
KQVQE--KPKEQAPVKTQET
---------KEQVPVKSQET
KQVQE--KPKEQAPVKSQET
KQVQQQQ-PKEQAPVKTQET
KQVQQQ--PKEQAPVKTQET
---------KEQAPVKTQET
KQVQHQ--PKEQAPVKAQEI
KQVQQQQ-PKEQAPVKAQET
KQVQQQ--PKEQAPVKTQET
KQVQHQ--PKEQAPVKAQET
KQVQQQQQPKEQAPVKTQET
KQVQE--KPKEQAPVKTQET
KHVQE--KPKEQAPVKTQET
KQVQQQ--PKEQAPVKTQET
KQVQQQ--PKEQAPVKTQET
KQVQQQ--PKEQAPVKTQET
KQVQE--KPKEQAPVKAQET
KQVQQQQ-PKEQAPVKTQET
KHVQE--KPKEQAPVKSQET
KQVQQQQ-PKEQAPVKTQET
KQVQQQQ-PKEQAPVKTQET
KQVQQQ--PKEQAPVKTQET
KQVQQQ--PKEQAPVKTQET
---------KEQAPVKSQET
KQVQE--KPKEQAPVKAQET
KQVQQQ--PKEQAPVKAQET
KQVQQQ--PKEQAPVKTQET
KQVQQQ--PKEQAPVKAQEA
KQVQQQQ-PKEQAPVKTQET
KQVQQQ--PKEQAPVKAQET
KQVQQQ--PKEQAPVKAQET
KQVQQQ--PKEQAPVKTQET
KQVQQQ--PKEQAPVKAQEA
KQVQQQQQPKEQAPVKSQET
KQVQQQ--PKEQAPVKAQET
KQVQQQQ-PKEQAPVKAQET
KQVQEKPKEPAKEPAAKKLF
```

Figure S2. Repeat organization of the amino acid sequence of *Rhinatrema bivitattum* SEDC2 underlying the sequence logo shown in Figure 2B. The amino acid sequence of *Rhinatrema bivitattum* SEDC2 is shown with lysine (K), glutamine (Q) and proline (P) being highlighted as in Figure S1. Line breaks and dashes were introduced to create an alignment of sequence repeats. SEDC, Simple epidermal differentiation complex gene. The alignment was used to generate a sequence logo using the weblogo tool at https://weblogo.berkeley.edu/logo.cgi, last accessed on 7 March, 2022. The logo is shown in Figure 2B.

```
>Xt_S100A16
KLESAIEVLVQNFYVYAEKKG--KQDKMNKKEFRKMVTQELQHVLTNTQNKEAANKLIQSLDADEDGKISFDEYWTLIGEIAKKLSMQM
>Am_S100A16
DLERSIEVLVKNFYQYA---G--KKEKLNRKEFRKMVGAELSHILTNTQSKEGADKLIKSLDANDDGKISFDEYWTLIGEIAKKLSQQM
>Mu_S100A16
ELEKSIEVLVRNFYKYAEKKG--KKDKMTKKEFRKMVGSELNHILTNTQSKEGADKLIKSLDADEDGKISFDEVLDLIVEIAKELSQAN
>Rb_S100A16
ELEKSIEVLVRNFYKYAERKG--KKDKMTKKEFRKMVSSELNHILTNTQSKEGADKLIKSLDADEDGKISFDEYWTLIGEIAKKLSQQI
>Gs_S100A16
ELERSIEVLVRNFYKYAEKKG--KKDKMTKKEFRKMVSSELSHILTNTQSKEGADKLIKSLDADEDGKISFDEYWTLIGEIAKKLSQQI
>Hs_S100A16
ELEKAVIVLVENFYKYVSKYSL-VKNKISKSSFREMLQKELNHMLSDTGNRKAADKLIQNLDANHDGRISFDEYWTLIGGITGPIAKLI
>Gg_S100A16
ELEWAVQVLVNNFDKYSSRCCCRKPRRISKKDFRKMLSRELNHMLTDTGNRRAADKLICDLDENKDGRISFEEYWTLIGGIASPIAHII
>Hs_filaggrin
TLLENIFAIINLFKQYSKKDK--NTDTLSKKELKELLEKEFRQILKNPDDPDMVDVFMDHLDIDHNKKIDFTEFLLMVFKLAQAYYEST
>Gg_Scaffoldin
HFLDSVSTIITVFYQHAKEDG--DQSKLNRRKMKEFIEKEFADAIVNPHDPQTIEKILQFLEWDGDGEIDFNEFLLLVFRVAKACYWYL
>Hs_trichohyalin
PLLRSICDITEIFNQYVSHDC--DGAALTKKDLKNLLEREFGAVLRRPHDPKTVDLILELLDLDSNGRVDFNEFLLFIFKVAQACYYAL
>Gg_cornulin
QLQENINGIITVFYTYARSDG--DSSTLSRGELRQLIEQEFGDVITDARDPRTVDEVLFFLDEDSSGKIDFGEFLSLIFRVAKACHRQL
>Hs_cornulin
QLLQNINGIIEAFRRYARTEG--NCTALTRGELKRLLEQEFADVIVKPHDPATVDEVLRLLDEDHTGTVEFKEFLVLVFKVAQACFKTL
>Xt_SFTP1
KLLAAVRGTIEAFNLYRSQSS--SCNSLSPEEFKRLIEKEFVDIIKESKQPETIKKVLQSLDTKSQKEVNFKDFMEIFKNVSVAYYDAS
>Am_SFTP2
SFMESIDNITTIFQKYASKEC--GRDKLNKAELTQLLQDELSPAMKVSGD-GSINTILKALDQNGDGQVDFNEFLVLVFKVAKAYNEHV
>Am_SFTP1
NLLRALKLIIDTFNSYSRQQG--HFDKLNKDEFNLLIQNEFADIIEDSENPQTIAAIMKALDENDDGEVDFKEFMALLFKVALAYYEAE
>Mu_SFTP1
RLLSAIKSVIDTYDSYCSKEQ--SHDKLNPEAFNRLIQNEFADVIEDSENPKTIEAIMQVLDENHDGEVDFREFLDLFFKVIVAYYDAL
>Gs_SFTP1
QLLNAIKSVIDTFENYCSKEQ--GYGKLNKAAFNRLIQSEFADVIEDSENPKTIEAIMQVLDENDDGEVDFREFLDLFFKVIVAYYDAL
>Rb_SFTP1
QLLTIIKDIINTFYSYCNKED--LHGKLNKEAFNLLIQNEFADIIEDSEKPETIAAIMQALDENNDGEVDFKEFLDLFFKVAVAYCEAL
>Xt_SFTP2
KLTGVINEIIGIFLSYSEGTC--APHKLNKNQMNRLIQNEFSDVIKNPKDPKTVEALIKVLDQNKDEEVDFDEFSDLLCKVLKAIYRAM
>Am_SFTP3
TIVSALQDIVDVFLRYTAQEC--DRDKLNRAELESIIQNEFTDVIKNPKDPQTPATMLIVLDEDKDGQVDFNEFMNLVCKVAKAYYKTF
>Rb_SFTP2
SIIGAIQDLIDVFHSYSKEQC--KL--LNKDELKSLLQKELANVIKNPNDPQTVETVMKILDDNQDGGVDFNEFTDLMCKIIKAYYNTF
>Mu_SFTP2
FLISAIQDIIEVYLSYAHREC--NLQKLNKEELKSLIQNEFADVIKNPNDPQTAVTVMKTLDDNRDGEVDFEEFMDLMSKVSKAYYKTF
>Gs_SFTP2
SLINVIEDTIQVYFKYSQRSC--NHQKLNNEELKSLIQNEFADVIKNPNDPQTAETVLKILDENKDGKVDFEEFMDLLAKVAKAYFKTF
```

Figure S3. Alignment of S100 domain sequences used for phylogenetic analysis. The S100 domains of SFTPs and S100A16 were aligned with MAFFT. The sequences are shown in fasta_aln format. Dashes were introduced to optimize the alignment. The complete amino acid sequences of SFTPs of amphibians are shown in Figure S1. GenBank accession numbers of other proteins: Rb S100A16, XP_029435257; Mu S100A16, XP_030042730; Gs S100A16, XP_033780380; Xt S100A16, XP_031747447; Homo sapiens S100A16, NP_001303936; Gg S100A16, XP_040508506; Hs Filaggrin, NP_002007; Hs Cornulin, NP_057274; Hs Trichohyalin, NP_009044; Gg Cornulin, NP_001165847; Gg Scaffoldin, NP_001338424. Am, *Ambystoma mexicanum*; Gg, *Gallus gallus*; Gs, *Geotrypetes seraphini*; Hs, *Homo sapiens*; Mu, *Microcaecilia unicolor*; Rb, *Rhinatrema bivittatum*; Xt, *Xenopus tropicalis*.

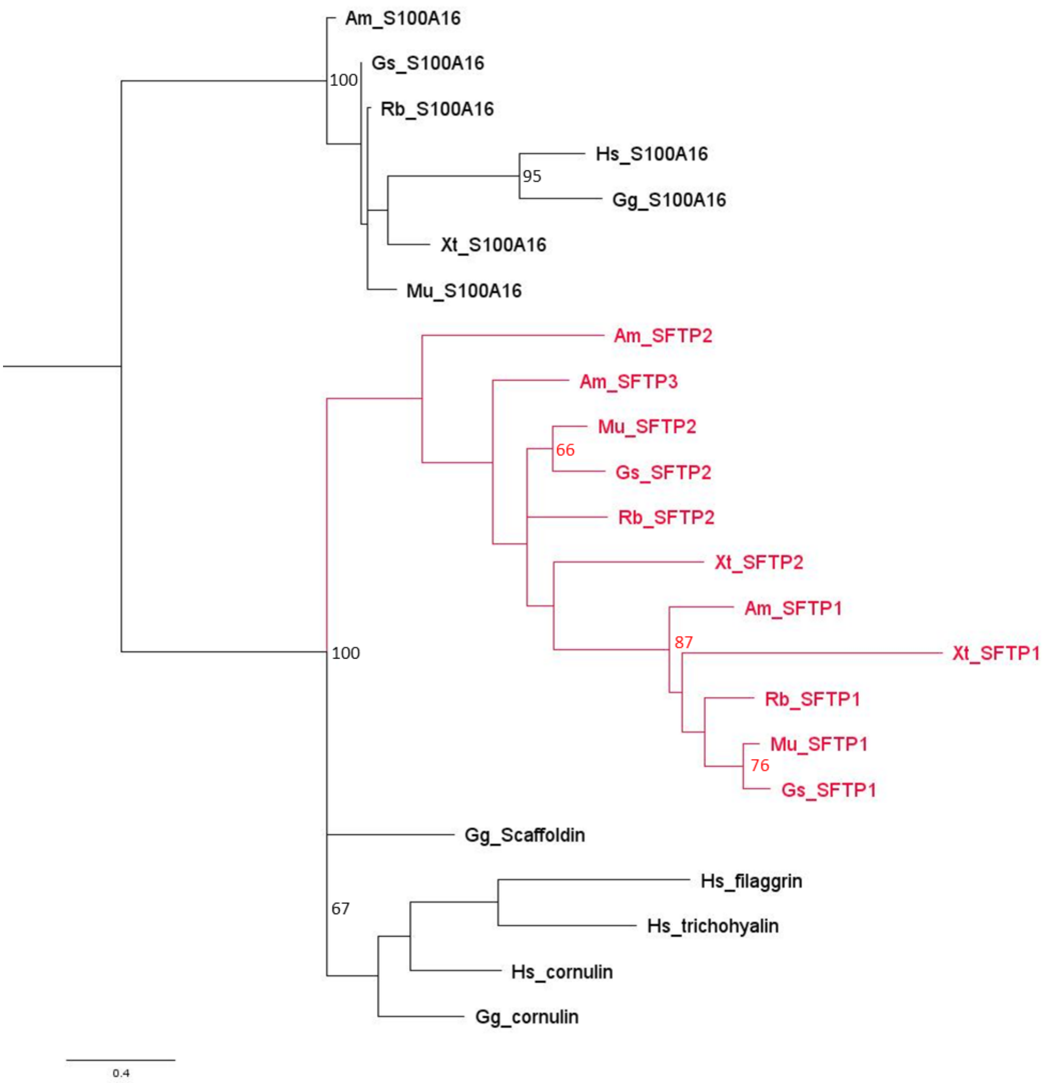

Figure S4. Phylogenetic analysis of SFTPs. The sequences of the S100 domains were aligned and used to construct the phylogenetic tree. S100A16 proteins were used as outgroup. Bootstrap values >60 are indicated. SFTP, S100 fused-type protein. Species: Am, *Ambystoma mexicanum*; Gg, *Gallus gallus*; Gs, *Geotrypetes seraphini*; Hs, *Homo sapiens*; Mu, *Microcaecilia unicolor*; Rb, *Rhinatrema bivittatum*; Xt, *Xenopus tropicalis*.

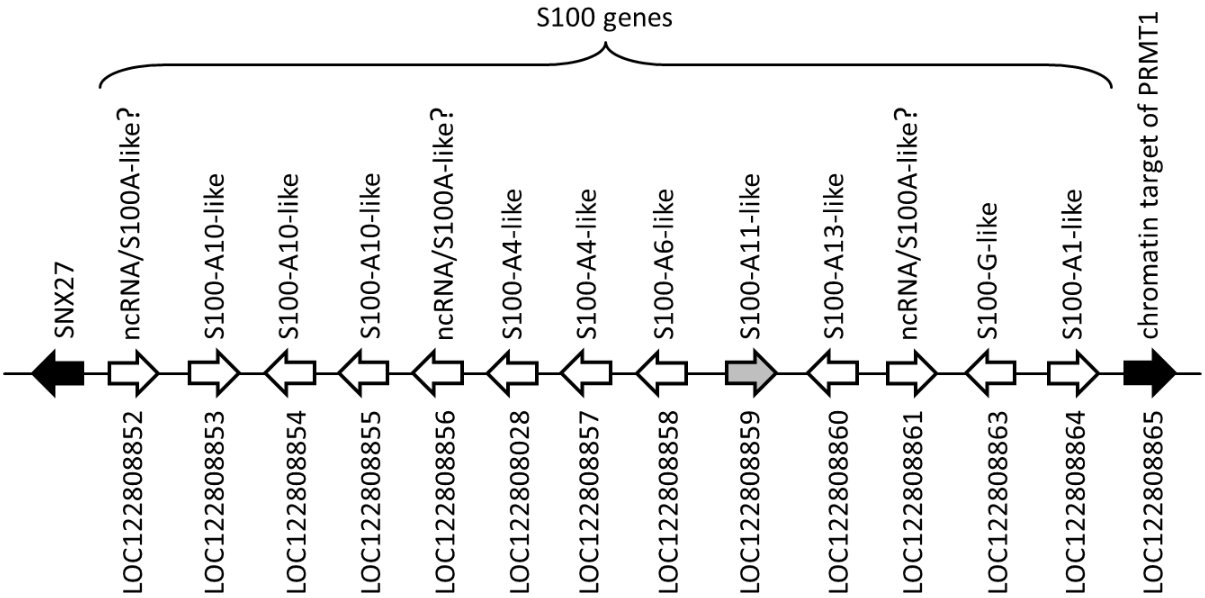

Figure S5. The S100A gene cluster on chromosome 8 of the African lungfish (*Protopterus annectens*) is predicted to contain neither *SEDC* nor *SFTP* genes. A segment of chromosome 8 of *Protopterus annectens*, corresponding to genome sequence assembly PAN1.0 (GCF_019279795.1), GenBank accesssion number NC_056741.1, nucleotide positions 430933198 – 444670046, was analyzed. Genes are depicted by arrows pointing in the direction of gene transcription. Annotations are from GenBank. Three genes are annotated as non-coding RNAs (ncRNAs) but their nucleotide sequences are similar to those of *S100A* genes. Note that no *SEDC* or *SFTP* genes are predicted in the region flanking the putative ortholog of *S100A11* (grey arrow). BLAST searches using SEDC proteins and SFTPs as queries were negative.

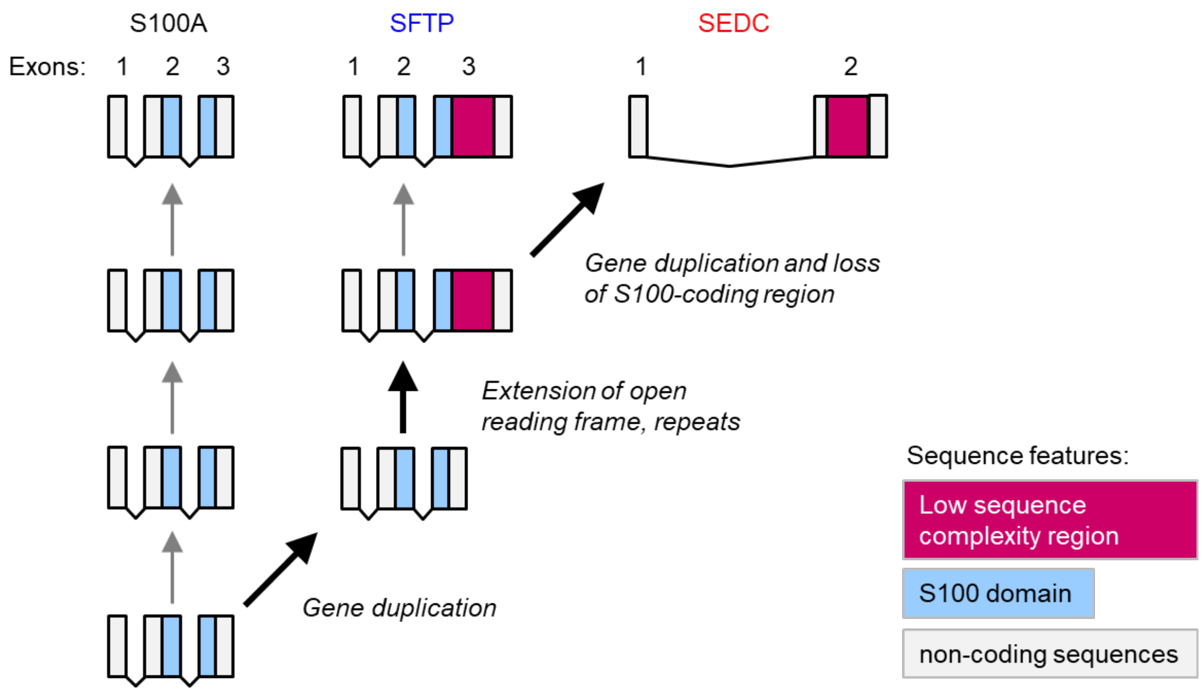

Figure S6. Evolutionary model for the origin of EDC gene types. The schematic depicts a hypothetical pathway of gene recombination and mutation events leading to the evolution of SFTP and SEDC genes from an S100A gene that was present in a fish ancestor of tetrapods. This model was developed from previous hypotheses (Strasser et al. 2014) taking into consideration the organisation of the EDC in newly available genome sequences and the absence of a *PGLYRP3* gene from the EDC of amphibians. Specifically, this model is a modification of scenario 4 of Supplementary Figure S13 in Reference Strasser et al. 2014.

Table S1. SEDC genes of caecilians

Species	Gene	Accession number (GenBank)	exon 1 end	exon 2 start	coding sequence start (exon 2)	coding sequence end (exon 2)	Orientation of gene	Confirmation by RNA-seq of skin	GenBank prediction of protein, accession number
Rhinatrema bivittatum	SEDC1	NC_042630.1	26179523	26189856	26189873	26190476	forward	yes	n.a.
Rhinatrema bivittatum	SEDC2	NC_042630.1	26289781	26291655	26291677	26297277	forward	yes	n.a.
Microcaecilia unicolor	SEDC1	NC_044044.1	37001319	36999093	36999073	36998210	reverse	yes	n.a.
Microcaecilia unicolor	SEDC2	NC_044044.1	36977283	36974970	36974887	36973724	reverse	yes	n.a.
Microcaecilia unicolor	SEDC3	NC_044044.1	36983767	37011504	37011524	37012123	forward	yes	XP_030043654.1
Microcaecilia unicolor	SEDC4	NC_044044.1	37037770	37038316	37038334	37041519	forward	yes	n.a.
Geotrypetes seraphini	SEDC1	NC_047099.1	25747663	25803662	25803682	25807008	forward	n.a.	XP_033781152.1
Geotrypetes seraphini	SEDC2	NC_047099.1	25784522	25781003	25780977	25779778	reverse	n.a.	XP_033781154.1

Abbreviations: RNA-seq, RNA sequencing; n.a., not available.

Table S2. SFTP genes of caecilians, axolotl and *Xenopus tropicalis*

Species	Gene	Accession number (GenBank)	exon 1 end	exon 2 start	coding sequence start (exon 2)	exon 2 end	exon 3 start	coding sequence end (exon 3)	Orientation of gene	GenBank prediction of protein, accession number
Rhinatrema bivittatum	SFTP1	NC_042630.1	26115567	26119185	26119200	26119337	26127989	26135020	forward	n.a.
Rhinatrema bivittatum	SFTP2	NC_042630.1	26161849	26164493	26164496	26164627	26168371	26171161	forward	n.a.
Microcaecilia unicolor	SFTP1	NC_044044.1	36896683	36902355	36902370	36902507	36910886	36922597	forward	n.a.
Microcaecilia unicolor	SFTP2	NC_044044.1	36954485	36958497	36958500	36958637	36961012	36962602	forward	XP_030043648.1
Geotrypetes seraphini	SFTP1	NC_047099.1	25955132	25940297	25940282	25940145	25926622	25911266	reverse	n.a.
Geotrypetes seraphini	SFTP2	NC_047099.1	25849649	25842556	25842541	25842404	25840504	25838156	reverse	n.a.
Ambystoma mexicanum	SFTP1	CM010927.2	n.a.	718074216	718074201	718074064	718047919	718047326	reverse	n.a.
Ambystoma mexicanum	SFTP2	CM010927.2	n.a.	717769809	717769804	717769667	717768256	717766007	reverse	n.a.
Ambystoma mexicanum	SFTP3	CM010927.2	n.a.	717517780	717517757	717517620	717516583	717515684	reverse	n.a.
Xenopus tropicalis	SFTP1	NC_030684.2	143977508	143969175	143969161	143969024	143963079	143950288	reverse	n.a.
Xenopus tropicalis	SFTP2	NC_030684.2	143901787	143897478	143897469	143897332	143896135	143893862	reverse	XP_031747944.1

Abbreviations: n.a., not available.
